# Meliacarpinin-Type Limonoids from the Bark of *Melia toosendan*

**DOI:** 10.3390/molecules23102590

**Published:** 2018-10-10

**Authors:** Yalin Hu, Li Heng, Rong Xu, Junhe Li, Shanshan Wei, Deran Xu, Jun Luo, Yi Li

**Affiliations:** 1 Testing & Analysis Center, Nanjing Normal University, Nanjing 210023, China; hengliyjs@163.com (L.H.); 18851138588@163.com (R.X.); 2 State Key Laboratory of Natural Medicines, Department of Natural Medicinal Chemistry, China Pharmaceutical University, 24 Tong Jia Xiang, Nanjing 210009, China; huyalin15@126.com (Y.H.); 15951718093@163.com (J.L.); 18851725100@163.com (S.W.); Dr-xu@163.com (D.X.); luojun1981ly@163.com (J.L.)

**Keywords:** *Melia toosendan*, limonoids, modified Mosher’s method, ECD exciton chirality, anti-inflammatory

## Abstract

Three new meliacarpinin-type limonoids, toosendanes A–C (1–3), along with three, known meliacarpinins (4–6) were isolated from the bark of *Melia toosendan*. Their structures, along with their absolute configurations, were elucidated, based on detailed analyses. These included HRESIMS and 1D/2D-NMR, modified Mosher’s method, and electronic circular dichroism (ECD). Limonoids 2 and 3 showed moderate inhibitory activity on LPS-activated, RAW 264.7 macrophages.

## 1. Introduction

**Figure 1:**
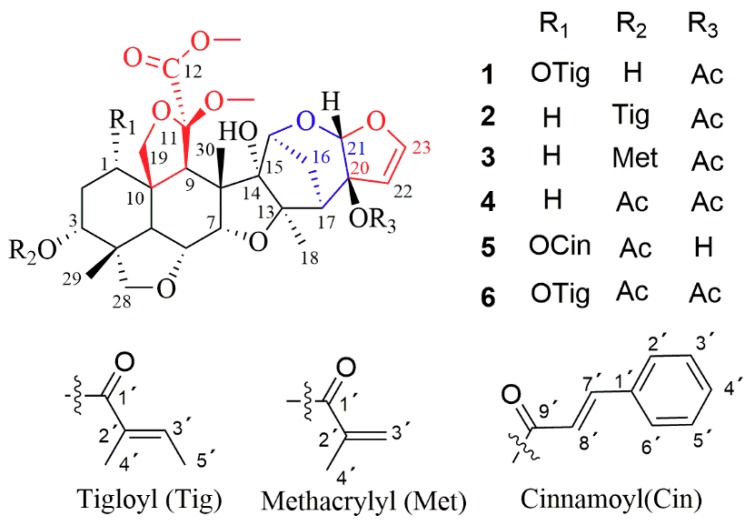
Chemical structures of limonoids 1–6.

*Melia toosendan* (Meliaceae) usually occurs as a large tree and is mainly distributed within the tropical and subtropical areas of Asia, including southern China (mainly in the Sichuan and Yunnan Provinces). Its fruits, known as toosendan fruits or chuan lian zi, are recorded in the Chinese Pharmacopoeia and have been commonly used in traditional Chinese medicines (TCMs) as painkillers and anthelmintics [1,2]. Many components—including euphane- and tirucallane-type triterpenoids and tetranortriterpenoids (such as trichilinins), ring C-seco limonoids, C-19/C-29-bridged acetals, meliacins, spiro limonoids, highly-oxidized, C-seco limonoids, and steroids—have been isolated from various parts of the plant [3,4,5,6,7,8,9,10,11]. Limonoids have been reported to exhibit cytotoxic, antibacterial, antifeedant, anti-inflammatory, analgesic, and growth-inhibitory activities [8,9,10,11,12,13]. Previously, we reported a series of triterpenoids, with diverse side-chains, from the bark of *M. toosendan* [14]. Continuation of this research afforded three, new, meliacarpinin-type limonoids (toosendanes A–C; 1–3), as shown in Figure 1, along with three known ones (4–6). Their structures, along with their absolute configurations, were established by spectroscopic methods, including HRESIMS, NMR, and modified Mosher’s method. All new limonoids were evaluated in vitro for their anti-inflammatory activity. Herein, we describe the isolation, identification, and bioactivity of the new limonoids.

## 2. Results and Discussion

Toosendane A (1) was obtained as a colorless, amorphous powder. Its molecular formula was determined as C_35_H_46_O_14_, from the [M + Na]^+^ peak at m/z 713.2783 (calcd. 713.2780). The IR spectrum showed absorption peaks at 3460 cm^−1^ (OH), 1742 cm^−1^ (C=O), and 1618 cm^−1^ (C=C). The ^1^H and ^13^C-NMR data, shown in Table 1, indicated the presence of three, tertiary methyls (*δ*_H_ 1.50, 1.34, 0.93) and two methoxyls (*δ*_H_ 3.70, 3.35), one disubstituted olefin group (*δ*_H_ 6.41, d, *J* = 3.0 Hz; 5.40, d, *J* = 3.0 Hz; *δ*_C_ 146.9, 105.7), two acetal carbons (*δ*_H_ 5.67, s; *δ*_C_ 107.0; 106.1), and two, downfield, oxygenated carbons (*δ*_C_ 93.8, 93.0). These observations indicated that toosendane A was possibly a meliacarpinin-type limonoid [15].

**Figure 2:**
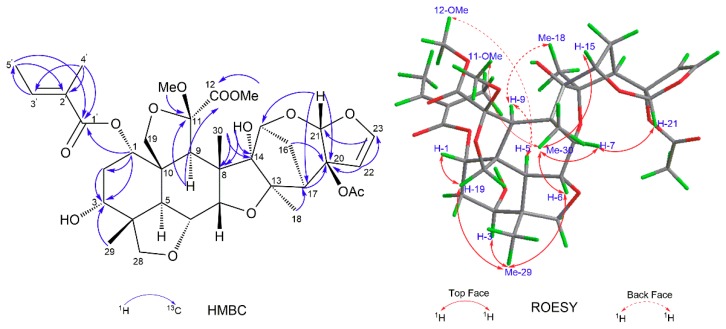
Key HMBC and ROESY correlations of compound **1**.

The NMR spectroscopic data of limonoid 1, as shown in Table 1, were similar to those of 1-tigloyl-3,20-diacetyl-11-methoxymeliacarpinin (limonoid 6) [15]. The main difference between these compounds was that there was only one acetyl group in limonoid 1, which was located at C-20 by the chemical shift of C-20 (*δ*_C_ 91.9) [15]. The obvious, upfield shifts of H-3, between limonoid 1 (*δ*_H_ 3.82) and limonoid 6 (*δ*_H_ 4.88), revealed that OH-3 in limonoid 1 was not acylated. This was also supported by the cross-peaks of C-3 (*δ*_C_ 70.4)/H-29 (*δ*_H_ 0.93, s) and H-1 (*δ*_H_ 4.88, t, *J* = 2.9 Hz) in the HMBC spectrum, as shown in Figure 2. Further, the 1-*O*-tigloyl group was proved by the cross-peak of C-1’ (*δ*_C_ 166.4)/H-1 (*δ*_H_ 4.88, t, *J* = 2.9 Hz); H-4’ (*δ*_H_ 1.84, s)/C-1’ (*δ*_C_ 166.4) and C-3’ (*δ*_C_ 138.8); H-5’ (*δ*_H_ 1.79, d, *J* = 7.1 Hz)/C-1’ (*δ*_C_ 166.4) and C-2’ (*δ*_C_ 128.3) in the HMBC spectrum. Thus, the planar structure of limonoid 1 was determined, as depicted in Figure 1.

**Figure 3:**
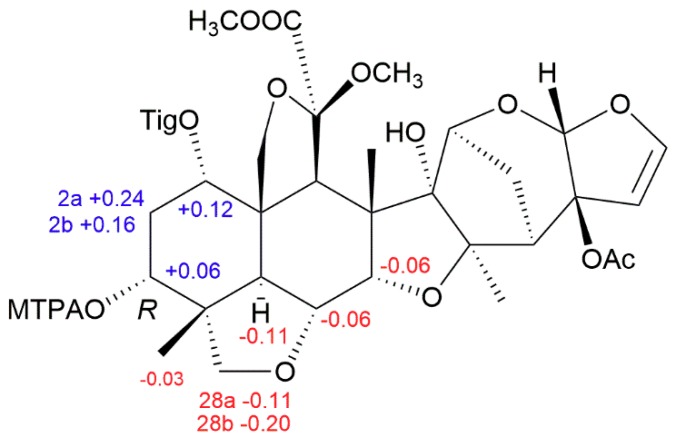
Determination of absolute configuration of limonoid 1 by modified Mosher’s method.

The relative configuration of limonoid 1 was determined to be the same as that of limonoid 6, through a ROESY experiment [15]. As shown in Figure 2, the ROESY correlations between H-5/H-9, H-5/12-OMe, and Me-18/H-9 showed that H-5, H-9, Me-18, and 12-OMe were on the same face and arbitrarily assigned as α-oriented, whereas the ROESY interactions between Me-30/H-6, Me-30/H-7, Me-30/11-OMe, Me-29/H-3, H-19 (*δ*_H_ 3.85, t, *J* = 9.2 Hz)/H-1, H-19 (*δ*_H_ 3.85, t, *J* = 9.2 Hz)/Me-29, Me-29/H-6, H-21/H-7, and Me-30/H-15 suggested the β-orientation of H-1, H-3, H-6, H-7, H-15, H-21, Me-29, Me-30, and 11-OMe. Because of the presence of free OH-3, the absolute configuration of limonoid 1 could be determined by modified Mosher’s method [16]. Treatment of limonoid 1 with (R)- and (S)-α-methoxy-α-(trifluoromethyl)-phenylacetyl chloride (MTPA-Cl) gave the S- and R-MTPA esters of limonoid 1 (limonoids 1a and 1b, respectively**)**. The ^1^H NMR signals of the two, MTPA esters were assigned on the basis of their ^1^H-^1^H ROESY spectra. The Δ*δ*_H_ (S–R) values were calculated, as shown in Figure 3. Based on these data, the absolute configuration of C-3 in limonoid 1 was determined to be *R*. Therefore, the absolute configurations of the other chiral carbons were determined, as shown in Figure 3, and the structure of limonoid 1, fully determined.

Toosendane B (limonoid 2), a colorless, amorphous powder, gave the molecular formula of C_35_H_46_O_13_, as established by an HRESIMS ion at *m*/*z* 697.2834, [M + Na]^+^ (C_35_H_46_NaO_13,_ calculated as 697.2831), indicating 13 degrees of unsaturation. The NMR spectrum of limonoid 2 showed close similarity to that of limonoid 1 and 3, 20-diacetyl-11-methoxymeliacarpinin (limonoid 4) [17], with the main difference being found in ring A. Detailed analysis of the HMBC correlations of ring A enabled us to determine the structure of limonoid 2. Key cross-peaks between H-3 (*δ*_H_ 4.99, t, *J* = 2.7 Hz)/C-1’ (*δ*_C_ 167.2) and C-1 (*δ*_C_ 33.7), Me-29 (*δ*_H_ 0.97, s)/C-3 (*δ*_C_ 70.9) indicated that the -*O*-tigloyl group was located at C-3. In addition, a ROESY correlation of Me-29/H-3 indicated the α-orientation of the 3-*O*-tigloyl group. The other, relative configuration was shown to be the same as that of limonoid 1. The similar, electronic circular dichroism (ECD) spectra revealed that the absolute configuration of limonoid 2 was consistent with that of limonoid 1. Thus, limonoid 2 was discovered and named toosendane B.

Toosendane C (limonoid 3) was isolated as a colorless, amorphous powder. Its molecular formula was C_34_H_44_O_13_, deduced from the HRESIMS spectrum at *m*/*z* 683.2673 [M + Na]^+^ (C_34_H_44_NaO_13,_ calculated as 683.2674) (see Supplementary Materials). The NMR spectra were similar to those of limonoid 2**,** except for an O-methacrylate group (*δ*_H_ 6.13, s; 5.62, t, *J* = 1.6 Hz; 1.96, br s; *δ*_C_ 166.5; 136.4; 126.2; 18.3) replacing the 3-tigloyloxyl group of limonoid 2. This conclusion was further confirmed by the following key HMBC correlations: H-3’ (*δ*_H_ 6.13, s)/C-2’ (*δ*_C_ 136.4) and C-1’ (δ_C_ 166.5), Me-4’ (*δ*_H_ 1.96, br s)/C-1’ (*δ*_C_ 166.5), C-2’ (δ_C_ 136.4) and C-3’ (δ_C_ 126.2), and Me-29 (*δ*_H_ 0.98, s)/C-3 (*δ*_C_ 71.3). The relative configuration of limonoid 3 was determined to be the same as that of limonoid 2, through its similar ROESY spectrum. The absolute configuration of limonoid 3 could not be determined, based on current information. On the basis of these results, the structure of limonoid 3 was identified and represented in Figure 1.

Three known compounds were identified as 3, 20-diacetyl-11-methoxymeliacarpinin (limonoid 4) [17], 3-acetyl-1-cinnamoyl-11-methoxymeliacarpinin (limonoid 5) [15], and 3,20-diacetyl-1-tigloyl-11-methoxymeliacarpinin (limonoid 6) [15], through comparison of their spectroscopic data (MS and NMR) with reported values.

All new compounds were evaluated for their inhibitory activity on LPS-activated, RAW 264.7 macrophages with *N*-Monomethyl-l-arginine (l-NMMA) (IC_50_: 41.9 ± 0.9 μM) as the positive control. Limonoids 2 and 3 showed moderate inhibitory effects with IC_50_ values of 21.3 ± 1.5 μM and 20.7 ± 2.3 μM, respectively.

## 3. Materials and Methods

### 3.1. General

Analytical HPLC was carried out, using an Agilent 1200 Series instrument (Agilent Technologies, Santa Clara, CA, USA), with a DAD detector that used a Shim-pack VP-ODS column (250 × 4.6 mm, 5 µm). Semi-preparative HPLC was performed on a Shimadzu LC-6A instrument with a shim-pack RP-C18 column (20 × 200 mm, 10 µm) and a SPD-20A detector. Silica gel (Qingdao Haiyang Chemical Co., Ltd. Qingdao, China), MCI gel (Mitsubishi Chemical Corp., Tokyo, Japan), Sephadex LH-20 (Pharmacia, Germany), and RP-C18 (40–63 µm, FuJi, Japan) were used for column chromatography. HRESIMS was performed on an Agilent UPLC-Q-TOF (Agilent Technologies, Santa Clara, CA, USA) mass spectrometer (6520 B). UV spectra were obtained on a Shimadzu UV-2450 spectrophotometer (Shimadzu, Tokyo, Japan). IR spectra were acquired on KBr discs, using a Bruker Tensor 27 spectrometer (Bruker, Karlsruhe, Germany). A JASCO P-1020 polarimeter (Jasco, Tokyo, Japan) was used for measuring the optical rotations. 1D and 2D NMR spectra (500 MHz (^1^H) and 125 MHz (^13^C)) were recorded on a Bruker AVIII-500 NMR spectrometer (Bruker, Karlsruhe, Germany) with TMS as the internal standard in CDCl_3_. CD spectra were obtained on a JASCO 810 spectrometer (Jasco, Tokyo, Japan).

### 3.2. Plant Material

The air-dried root and stem bark of *Melia toosendan* Sieb. et Zucc. were collected in Jiangyou, Sichuan province, the People’s Republic of China, in July 2015, and identified by Mian Zhang, a professor at the Research Department of Pharmacognosy of China Pharmaceutical University. A voucher specimen (2015-MMTA) was deposited in the Department of Natural Medicinal Chemistry, China Pharmaceutical University (Nanjing, China).

### 3.3. Extraction and Isolation

A total of 4.7 kg of dried bark from *M. toosendan* was extracted by reflux with 95% ethanol, three times, at 90 °C (3 h × 3). The EtOH extract was concentrated under reduced pressure, then suspended in H_2_O, and partitioned with petroleum ether and CH_2_Cl_2_. The CH_2_Cl_2_ extract (105 g) was loaded into a silica gel column, eluted with CH_2_Cl_2_-MeOH at a gradient of 100:1 to 0:100 *v*/*v*, which gave seven fractions (Frs. A–G). These were monitored by TLC and HPLC analysis. Fr. C (7.3 g) was chromatographed on a column of silica gel, eluted with a gradient of petroleum ether/acetone (10:1 to 2:1 *v*/*v*), to give three sub-fractions (Frs. C1–3). Fr. C2 (2.1 g) was chromatographed on a RP-C18 column, eluted with MeOH/H_2_O (20:80 to 100:0), to give five sub-fractions (Frs. C2a–e). Fr. C2b (30 mg) was separated by semi-preparative HPLC, using MeOH/H_2_O (45:55 *v*/*v*, 10 mL/min) as the mobile phase, to give toosendane A (27 mg). Fr. C2d (680 mg) was separated by semi-preparative HPLC, using MeOH/H_2_O (68:32 *v*/*v*, 10 mL/min) as the mobile phase to yield 3, 20-diacetyl-11-methoxymeliacarpinin (limonoid 4; 3 mg). Fr. D (6.2 g) was subjected to a MCI gel column (MeOH-H_2_O, 30:70 to 0:100 *v*/*v*), to afford five sub-fractions (Frs. D1–5). Fr. D2 (2.3 g) was subjected to an RP-C18 column, with a step gradient of MeOH-H_2_O (40:60 to 100:0 *v*/*v*), to give five sub-fractions (Fr. D2a–e). Fr. Db (1.1 g) was separated by semi-preparative HPLC, with MeOH-H_2_O (32:68 *v*/*v*, 10 mL/min), to give toosendane B (4 mg) and toosendane C (2.2 mg). Fr. E (9.8 g) was separated by silica gel and a Sephadex LH-20 gel column, to give six sub-fractions (Frs. E1–6). Fr. E3 (510 mg) was separated by an RP-C18 column, using a gradient of CH_3_OH/H_2_O (50:50 to 100:0 *v*/*v*) as the mobile phase, to afford three sub-fractions (Frs. E3a–c). Fr. E3a (80 mg) was separated by semi-preparative HPLC, using CH_3_OH/H_2_O (50:50, 10 mL/min) as the mobile phase, to give 3-acetyl-1-cinnamoyl-11-methoxymeliacarpinin (2.4 mg). Using the same purification procedure, Fr. E3c (70 mg) gave 3,20-diacetyl-1-tigloyl-11-methoxymeliacarpinin (3.7 mg).

Toosendane A: a colorless, amorphous powder; [α${]}_{D}^{25}$ − 14.1 (c 0.09, MeOH); UV (MeOH) λmax (log ε) 211 (3.98) nm; ECD (MeOH, Δε) 213 (−33.259), 238 (+9.704) nm; IR (KBr) λmax 3460, 2953, 1742, 1618, 1381, 1255, 1057 cm^−1^; ^1^H and ^13^C NMR data, as shown in Table 1; HRESIMS *m*/*z* 713.2783 [M + Na]^+^ (calculated for C_35_H_46_NaO_14_ as 713.2780).

Toosendane B: a colorless, amorphous powder; [α${]}_{D}^{25}$ − 10.5 (c 0.08, MeOH); UV (MeOH) λmax (log ε) 210 (3.87) nm; ECD (MeOH, Δε) 209 (−22.230), 236 (+2.774) nm; IR (KBr) λmax 3480, 2940, 1747, 1647, 1383, 1254, 1064 cm^−1^; ^1^H and ^13^C NMR data, as shown in Table 1; HRESIMS *m*/*z* 697.2834 [M + Na]^+^ (calculated for C_35_H_46_NaO_13_ as 697.2831).

Toosendane C: a colorless, amorphous powder; [α${]}_{D}^{25}$ − 14.6 (c 0.13, MeOH); UV (MeOH) λmax (log ε) 207 (3.88) nm; ECD (MeOH, Δε) 211 (−0.071), 224 (+3.472) nm; IR (KBr) λmax 3479, 2942, 1733, 1641, 1383, 1251, 1063 cm^−1^; ^1^H and ^13^C NMR data, as shown in Table 1; HRESIMS *m*/*z* 683.2673 [M + Na]^+^ (calculated for C_34_H_44_NaO_13_ as 683.2674).

### 3.4. Preparation of the (S)- and (R)-MTPA Esters of Limonoid 1

Dimethylaminopyridine (2 mg) and (*R*)-(-)-α-methoxy-α-(trifluoromethyl)-phenylacetyl chloride ((R)-(-)MTPA-Cl) (3 µL) were added to a solution of limonoid 1 (1 mg) in dried pyridine (100 µL). The mixture was allowed to stand overnight, at 30 °C. MeOH (2 mL) was added to quench the reaction and the reaction mixture was purified by preparative HPLC, using MeOH/H_2_O (95: 5 *v*/*v*) to give the pure (S)-MTPA ester (0.9 mg). The (*R*)-MTPA ester was prepared, using the method described above.

Limonoid 1a: HRESIMS *i* 929.3177 [M + Na]^+^ (calculated for C_45_H_53_F_3_NaO_16_ as 929.3178); ^1^H-NMR (CDCl_3_, 500 MHz): *δ*_H_ 5.21 (1H, t, *J* = 3.0 Hz, H-1), 4.77 (1H, t, *J* = 2.9 Hz, H-3), 4.22 (1H, d, *J* = 2.7 Hz, H-7), 3.93 (1H, dd, *J* = 12.7, 2.8 Hz, H-6), 3.54 (1H, d, *J* = 7.9 Hz, H-28a), 3.45 (1H, m, H-28b), 2.97 (1H, d, *J* = 12.8 Hz, H-5), 2.35 (1H, m, H-2a), 2.25 (1H, m, H-2b), and 1.05 (3H, s, Me-29).

Limonoid 1b: HRESIMS m/z 929.3179 [M + Na]^+^ (calculated for C_45_H_53_F_3_NaO_16_ as 929.3178); ^1^H-NMR (CDCl_3_, 500 MHz): *δ*_H_ 5.09 (1H, br s, H-1), 4.71 (1H, br s, H-3), 4.28 (1H, br s, H-7), 3.99 (1H, d, *J* = 12.7 Hz, H-6), 3.65 (1H, m, H-28a), 3.65 (1H, m, H-28b), 3.08 (1H, d, *J* = 12.6 Hz, H-5), 2.11 (1H, m, H-2a), 2.09 (1H, m, H-2b), and 1.08 (3H, s, Me-29). 

### 3.5. Anti-Inflammatory Activity

Anti-inflammatory activity was evaluated through the inhibition of lipopolysaccharide-(LPS) induced nitric oxide (NO) production in a macrophage (RAW264.7) cell line, which was obtained from the Chinese Academy of Science Cell Bank (Shanghai, China), as previously described [18]. All experiments were conducted in three, independent replicates, with *N*-Monomethyl-l-arginine (l-NMMA) as a positive control.

## Figures and Tables

**Table 1:** ^1^H-NMR (500 MHz) and ^13^C NMR (125 MHz) data of limonoids 1–3 in CDCl_3_ (*δ* in ppm, *J* in Hz).

Position	1		2		3	
	δ_H_	δ_C_	δ_H_	δ_C_	δ_H_	δ_C_
1	4.88 t (2.9)	71.9	1.39 m	33.7	1.38 m	33.6
			1.60 m		1.60 m	
2	2.09 m	31.0	1.84 ^a^ m	24.9	1.84 m	24.9
	2.18 m		1.87 m		1.86 m	
3	3.82 br s	70.4	4.99 t (2.7)	70.9	5.00 t (2.7)	71.3
4		43.9		43.0		42.9
5	3.05 d (12.6)	34.0	2.89 d (12.8)	39.4	2.85 d (12.8)	39.4
6	3.98 dd (12.6, 2.8)	71.9	3.90 dd (12.7, 2.7)	71.9	3.90 dd (12.7, 2.8)	71.9
7	4.27 d (2.7)	83.2	4.28 d (2.6)	83.6	4.28 d (2.7)	83.5
8		52.3		52.1		52.1
9	3.44 s	48.5	3.07 s	55.0	3.07 s	54.9
10		50.4		46.5		46.4
11		107.0		106.9		106.8
12		169.2		170.3		170.2
13		93.8		93.6		93.6
14		93.0		92.7		92.8
15	4.12 s	82.2	4.13 s	82.1	4.13 s	82.1
16	1.84 ^a^ m	29.0	1.83 m	29.1	1.82 m	29.1
	2.07 m		2.10 ddd (14.0, 6.3, 2.4)		2.10 ddd (13.9, 6.3, 2.4)	
17	2.94 d (5.9)	48.3	2.97 d (6.1)	48.3	2.96 d (5.4)	48.2
18	1.34 s	26.1	1.36 s	25.8	1.34 s	25.8
19	3.85 d (9.2)	70.9	3.95 d (8.5)	71.4	3.96 d (8.4)	71.3
	4.17 d (9.2)		4.06 d (8.5)		4.07 d (7.5)	
20		91.9		91.9		91.9
21	5.67 s	106.1	5.66 s	106.2	5.66 s	106.2
22	5.40 d (3.0)	105.7	5.38 d (3.0)	105.7	5.40 d (3.0)	105.7
23	6.41 d (3.0)	146.9	6.41 d (2.9)	146.9	6.41 d (3.0)	146.9
28	3.56 d (7.4)	76.2	3.48 d (7.8)	76.1	3.49 m	76.1
	4.03 d (7.4)		3.49 d (7.8)		3.51 m	
29	0.93 s 3H	18.7	0.97 s	18.5	0.98 s	18.5
30	1.50 s 3H	18.3	1.47 s	17.4	1.48 s	17.4
11-OCH_3_	3.35 s 3H	52.4	3.40 s	52.4	3.40 s	52.4
12-OCH_3_	3.70 s 3H	53.2	3.80 s	52.8	3.80 s	52.9
20-OAc	2.15 s 3H	21.7	2.02 s	21.3	2.03 s	21.3
		171.6		171.5		171.5
1’		166.4		167.2		166.5
2’		128.3		128.7		136.4
3’	6.86 m	138.8	6.89 dt (10.1, 3.6)	138.3	5.62 t (1.6)	126.2
					6.13 s	
4’	1.84 ^a^ s 3H	12.4	1.84 ^a^ s	12.1	1.96 br s	18.3
5’	1.79 d (7.1)	14.6	1.84 ^a^ s	14.6		
14-OH	4.14 s		4.17 s		4.17	

^a^ Overlapped.

## Supplementary Materials

The following are available online. Figure S1: HRESIMS spectrum of toosendane A; Figure S2: ^1^H-NMR (500 MHz, CDCl_3_) spectrum of toosendane A; Figure S3: ^13^C NMR (125 MHz, CDCl_3_) spectrum of toosendane A; Figure S4: HSQC spectrum of toosendane A; Figure S5: HMBC spectrum of toosendane A; Figure S6: ROESY spectrum of toosendane A; Figure S7: IR spectrum (KBr disc) of toosendane A; Figure S8: ECD spectra of toosendane A in MeOH; Figure S9. HRESIMS spectrum of 1a; Figure S10: ^1^H NMR (500 MHz, CDCl_3_) spectrum of limonoid 1a; Figure S11: ROESY spectrum of limonoid 1a; Figure S12. HRESIMS spectrum of 1b; Figure S13: ^1^H NMR (500 MHz, CDCl_3_) spectrum of limonoid 1b; Figure S14: ROESY spectrum of limonoid 1b; Figure S15: HRESIMS spectrum of toosendane B; Figure S16: ^1^H NMR (500 MHz, CDCl_3_) spectrum of toosendane B; Figure S17: ^13^C NMR (125 MHz, CDCl_3_) spectrum of toosendane B; Figure S18: HSQC spectrum of toosendane B; Figure S19: HMBC spectrum of toosendane B; Figure S20: ROESY spectrum of toosendane B; Figure S21: IR spectrum (KBr disc) of toosendane B; Figure S22: ECD spectra of toosendane B in MeOH; Figure S23: HRESIMS spectrum of toosendane C; Figure S24: ^1^H NMR (500 MHz, CDCl_3_) spectrum of toosendane C; Figure S25: ^13^C NMR (125 MHz, CDCl_3_) spectrum of toosendane C; Figure S26: HSQC spectrum of toosendane C; Figure S27: HMBC spectrum of toosendane C; Figure S28: ROESY spectrum of toosendane C; Figure S29: IR spectrum (KBr disc) of toosendane C; Figure S30: ECD spectra of toosendane C in MeOH); Figure S31. The NO inhibition rate and cell viabilities of toosendane B (2) and toosendane C (3) in different concentration.
