# Supplementary material for: Meliacarpinin-Type Limonoids from the Bark of Melia toosendan
Source: Molecules. 2018 Oct 10;23(10):2590. doi: 10.3390/molecules23102590 (PMC6222421; doi:10.3390/molecules23102590)

Supporting Information

Meliacarpinin-type limonoids from the bark of *Melia toosendan*

Yalin Hu[2†], Li Heng[1†], Rong Xu[1], Junhe Li[2], Shanshan Wei[2], Deran Xu[2], Jun Luo[2], Yi Li[1*]

1 Testing & Analysis Center, Nanjing Normal University, Nanjing 210023, China; hengliyjs@163.com (L.H.); 18851138588@163.com (R.X.)

2 State Key Laboratory of Natural Medicines, Department of Natural Medicinal Chemistry, China Pharmaceutical University, 24 Tong Jia Xiang, Nanjing 210009, China; huyalin15@126.com (Y.H.); 15951718093@163.com (J.L.); 18851725100@163.com (S.W.); Dr-xu@163.com (D.X.); luojun1981ly@163.com (J.L.)

* Correspondence: liyi16@163.com; Tel.: +86-25-83326900 (Y.L.)

† These authors contributed equally to this work.

List of Figures

Figure S1. HRESIMS spectrum of Toosendane A (**1**)

Figure S2. ^{1}H NMR (500 MHz, $CDCl_3$) spectrum of Toosendane A (**1**)

Figure S3. ^{13}C NMR (125 MHz, $CDCl_3$) spectrum of Toosendane A (**1**)

Figure S4. HSQC spectrum of Toosendane A (**1**)

Figure S5. HMBC spectrum of Toosendane A (**1**)

Figure S6. ROESY spectrum of Toosendane A (**1**)

Figure S7. IR spectrum (KBr disc) of Toosendane A (**1**)

Figure S8. ECD spectra of Toosendane A (**1**) (in MeOH)

Figure S9. HRESIMS spectrum of **1a**

Figure S10. ^{1}H NMR (500 MHz, $CDCl_3$) spectrum of **1a**

Figure S11. ROESY spectrum of **1a**

Figure S12. HRESIMS spectrum of **1b**

Figure S13. ^{1}H NMR (500 MHz, $CDCl_3$) spectrum of **1b**

Figure S14. ROESY spectrum of **1b**

Figure S15. HRESIMS spectrum of Toosendane B (**2**)

Figure S16. ^{1}H NMR (500 MHz, $CDCl_3$) spectrum of Toosendane B (**2**)

Figure S17. ^{13}C NMR (125 MHz, $CDCl_3$) spectrum of Toosendane B (**2**)

Figure S18. HSQC spectrum of Toosendane B (**2**)

Figure S19. HMBC spectrum of Toosendane B (**2**)

Figure S20. ROESY spectrum of Toosendane B (**2**)

Figure S21. IR spectrum (KBr disc) of Toosendane B (**2**)

Figure S22. ECD spectra of Toosendane B (**2**) (in MeOH)

Figure S23. HRESIMS spectrum of Toosendane C (**3**)

Figure S24. ^{1}H NMR (500 MHz, $CDCl_3$) spectrum of Toosendane C (**3**)

Figure S25. ^{13}C NMR (125 MHz, $CDCl_3$) spectrum of Toosendane C (**3**)

Figure S26. HSQC spectrum of Toosendane C (**3**)

Figure S27. HMBC spectrum of Toosendane C (**3**)

Figure S28. ROESY spectrum of Toosendane C (**3**)

Figure S29. IR spectrum (KBr disc) of Toosendane C (**3**)

Figure S30. ECD spectra of Toosendane C (**3**) (in MeOH)

Figure S31. The NO inhibition rate and cell viabilities of toosendane B (**2**) and toosendane C (**3**) in different concentration

Figure S1. HRESIMS spectrum of Toosendane A (**1**)

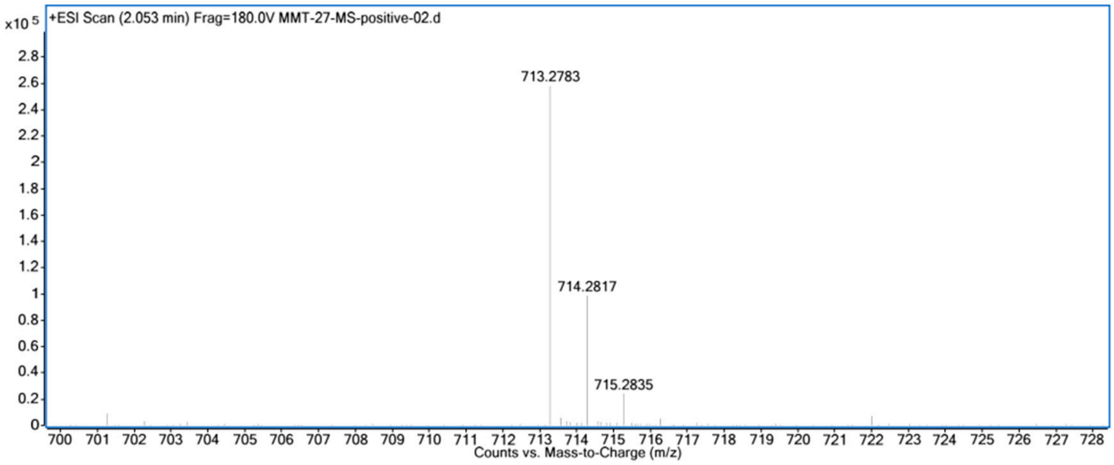

Elemental Composition Calculator

**Target m/z:**	713.2783	**Result type:**	Positive ions	**Species:**	[M+Na]+
**Elements:**		C (0-80); H (0-120); O (0-30); Na (0-5)			
**Ion Formula**		**Calculated m/z**		**PPM Error**	
C35H46NaO14		713.2780		-0.42	

Figure S2. ^{1}H NMR (500 MHz, $CDCl_3$) spectrum of Toosendane A (**1**)

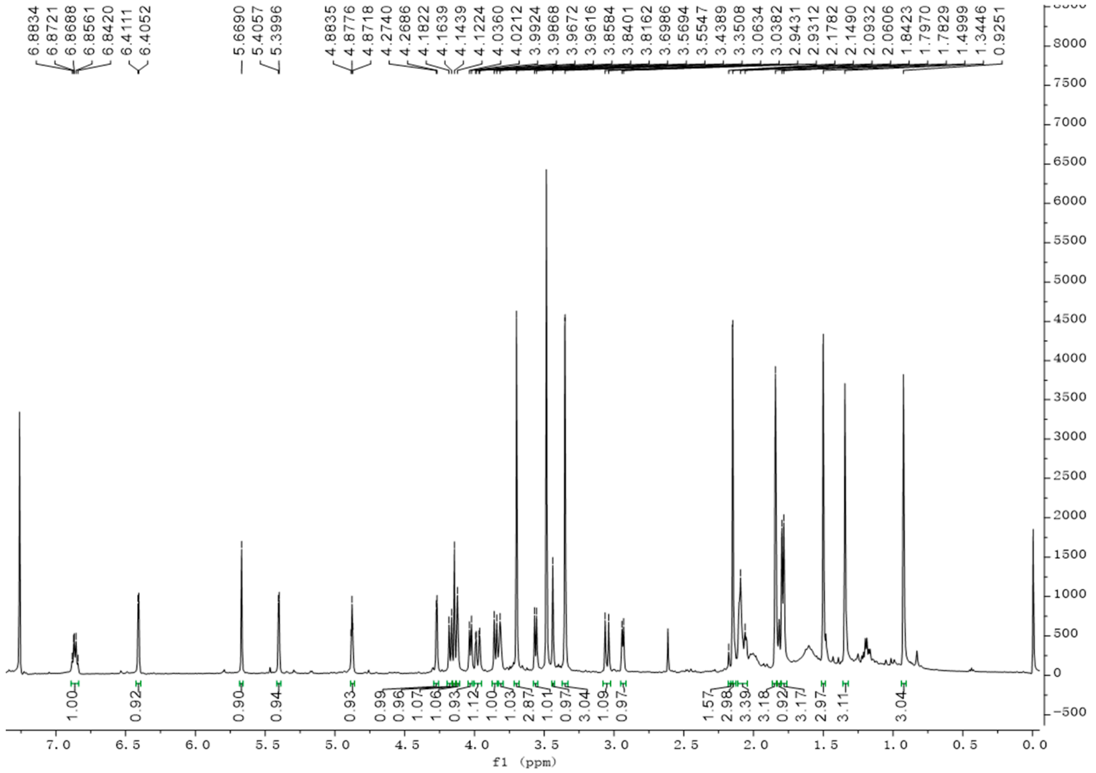

Figure S3. ^{13}C NMR (125 MHz, $CDCl_3$) spectrum of Toosendane A (**1**)

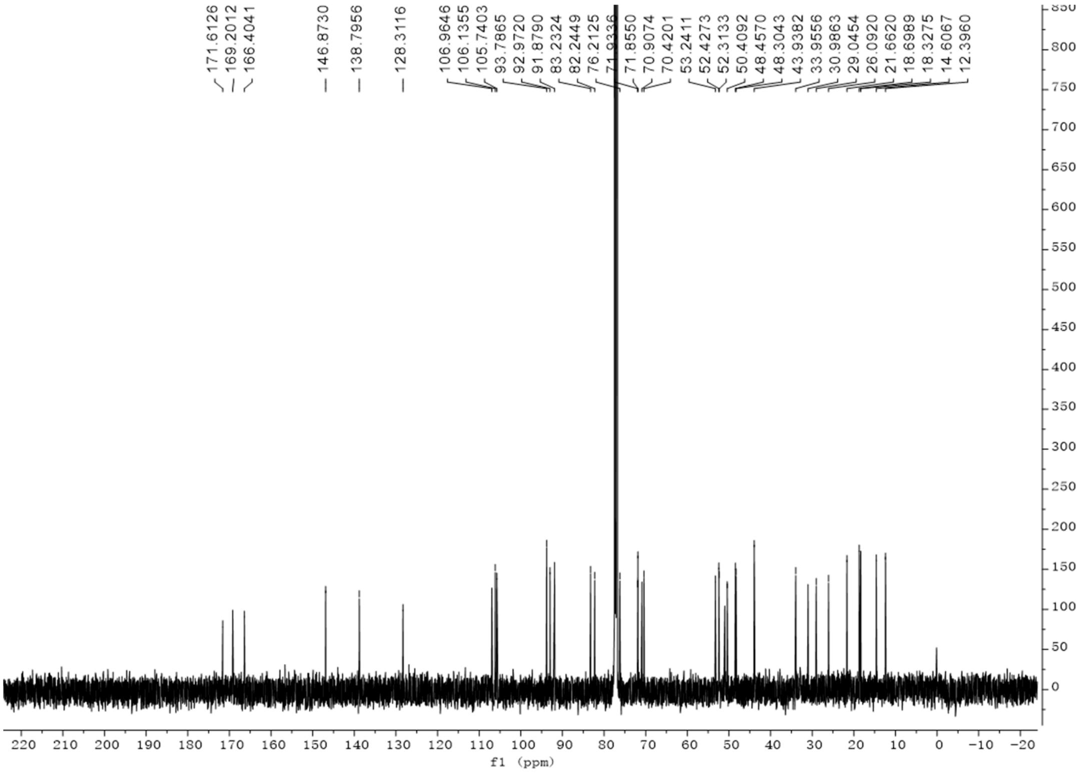

Figure S4. HSQC spectrum of Toosendane A (**1**)

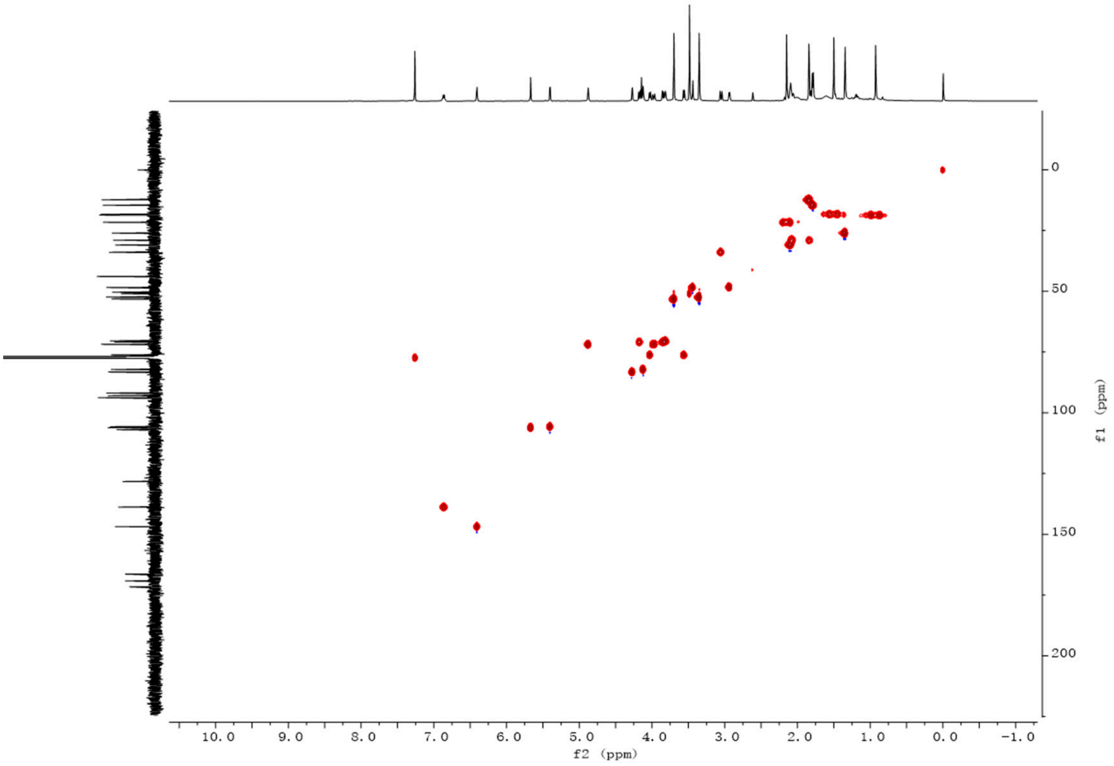

Figure S5. HMBC spectrum of Toosendane A (**1**)

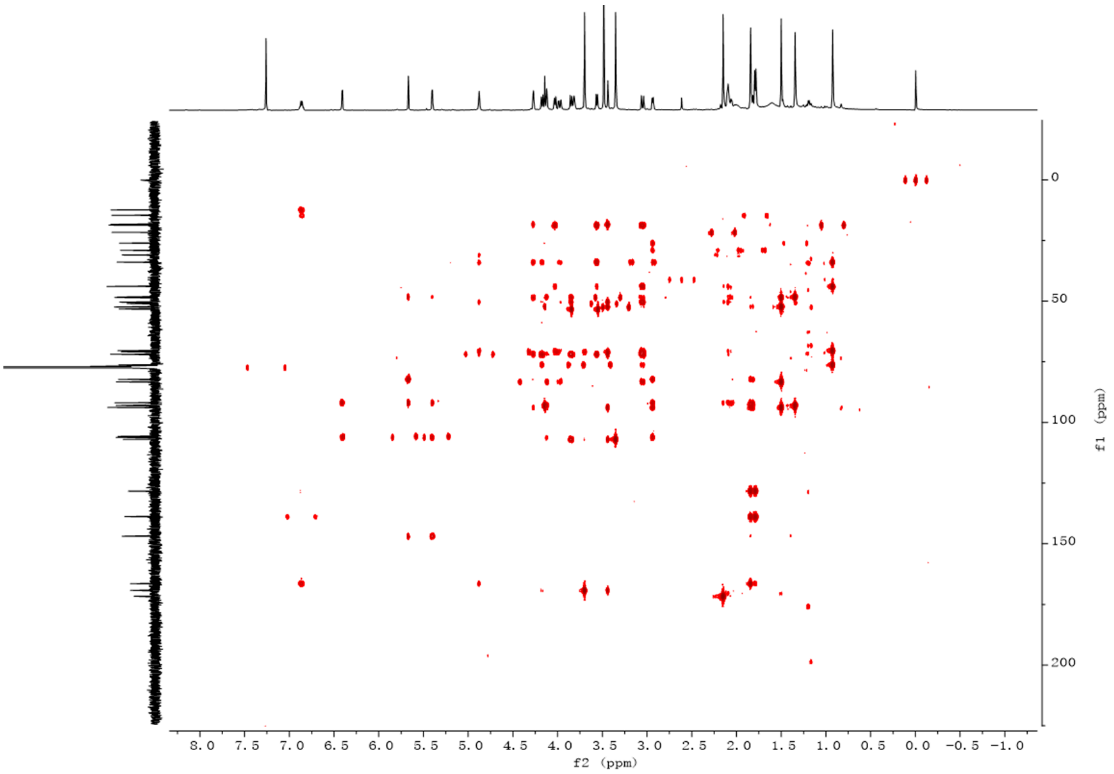

Figure S6. ROESY spectrum of Toosendane A (**1**)

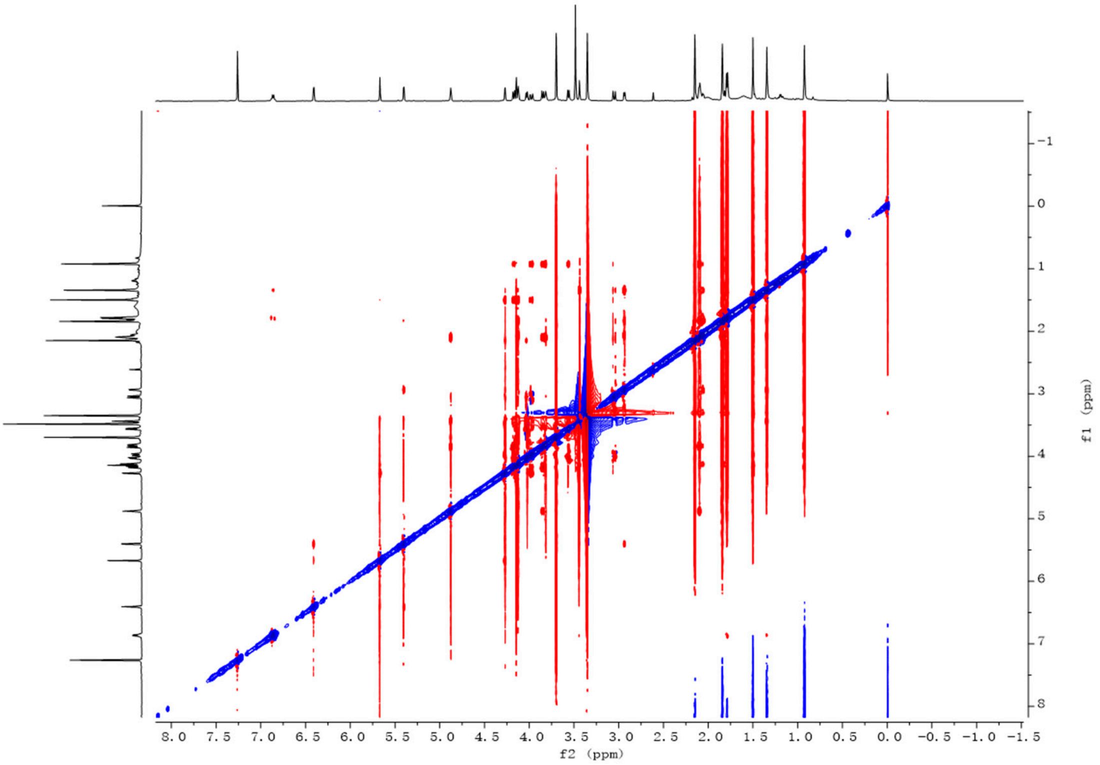

Figure S7. IR spectrum (KBr disc) of Toosendane A (**1**)

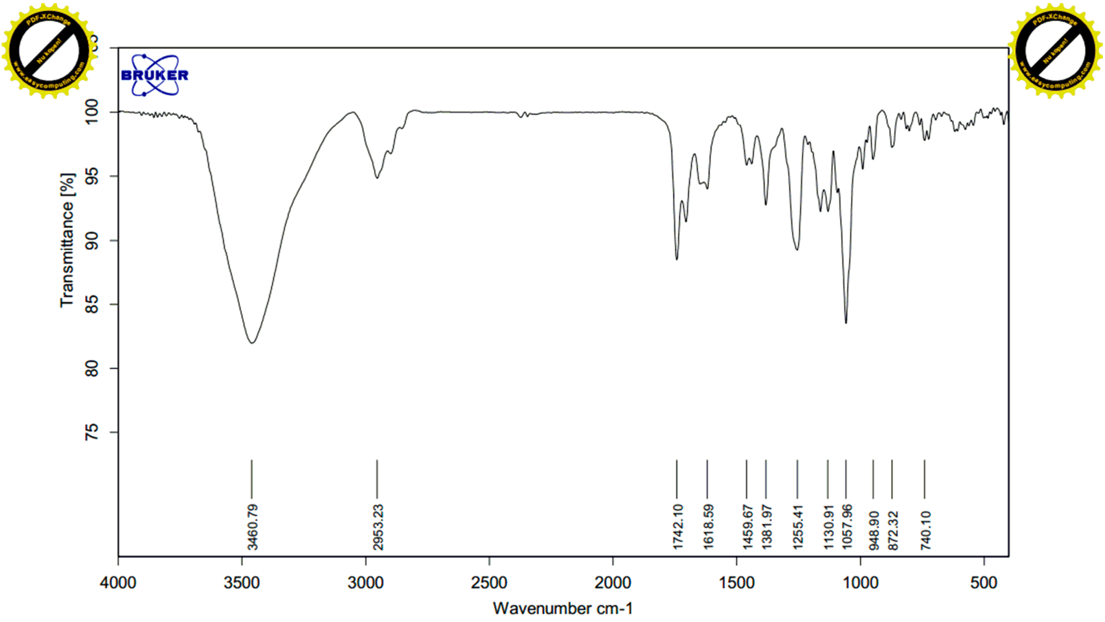

Figure S8. ECD spectra of Toosendane A (**1**) (in MeOH)

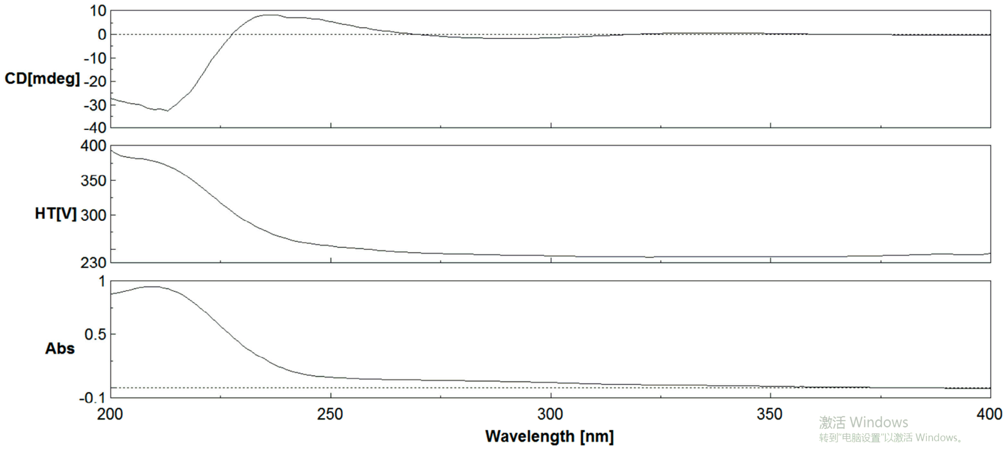

Figure S9. HRESIMS spectrum of **1a**

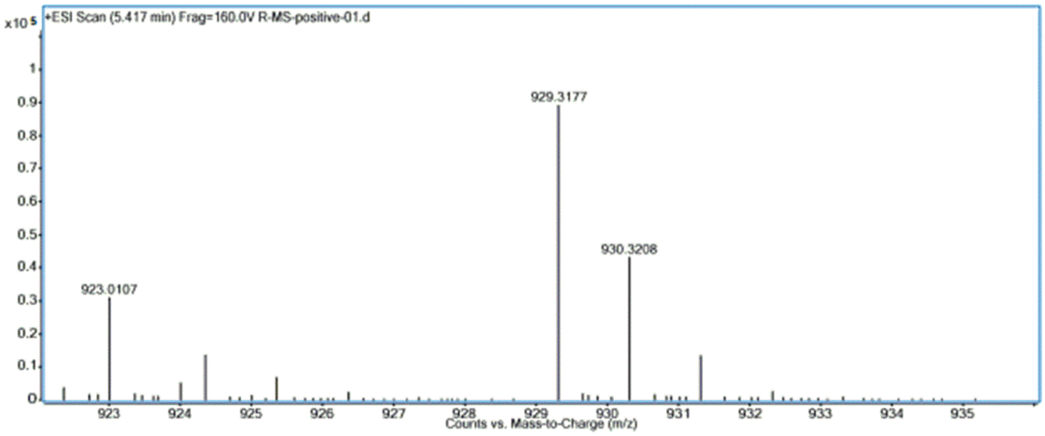

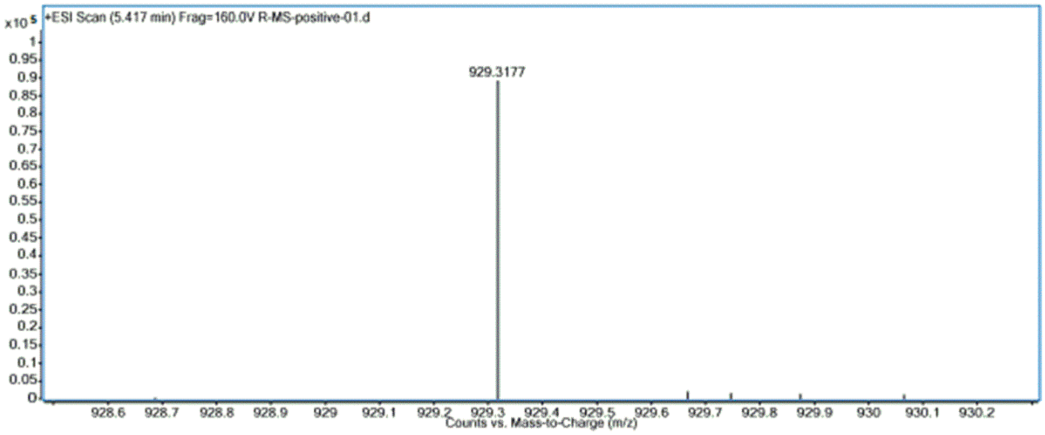

Elemental Composition Calculator

**Target m/z:**	929.3177	**Result type:**	Positive ions	**Species:**	$[M+Na]^+$
**Elements:**		C (0-80); H (0-120); O (0-30); Na (0-5)			
**Ion Formula**		**Calculated m/z**		**PPM Error**	
C45H53F3NaO16		929.3178		0.05	

Figure S10. ^{1}H NMR (500 MHz, $CDCl_3$) spectrum of **1a**

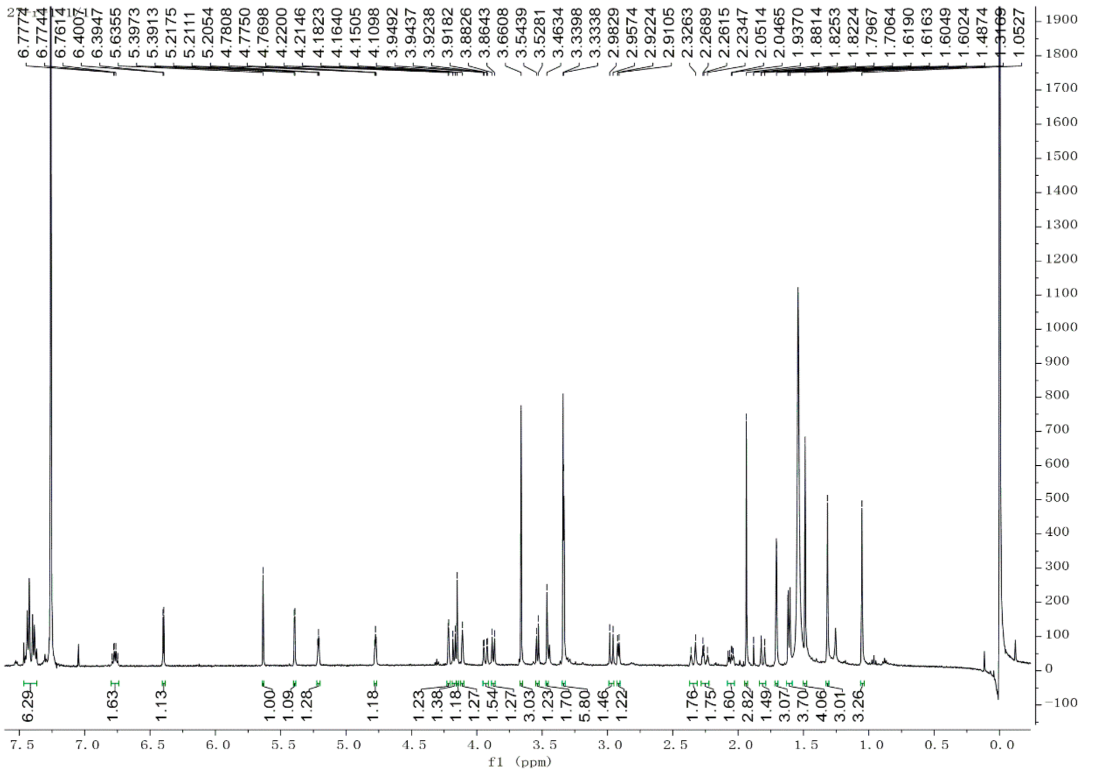

Figure S11. ROESY spectrum of **1a**

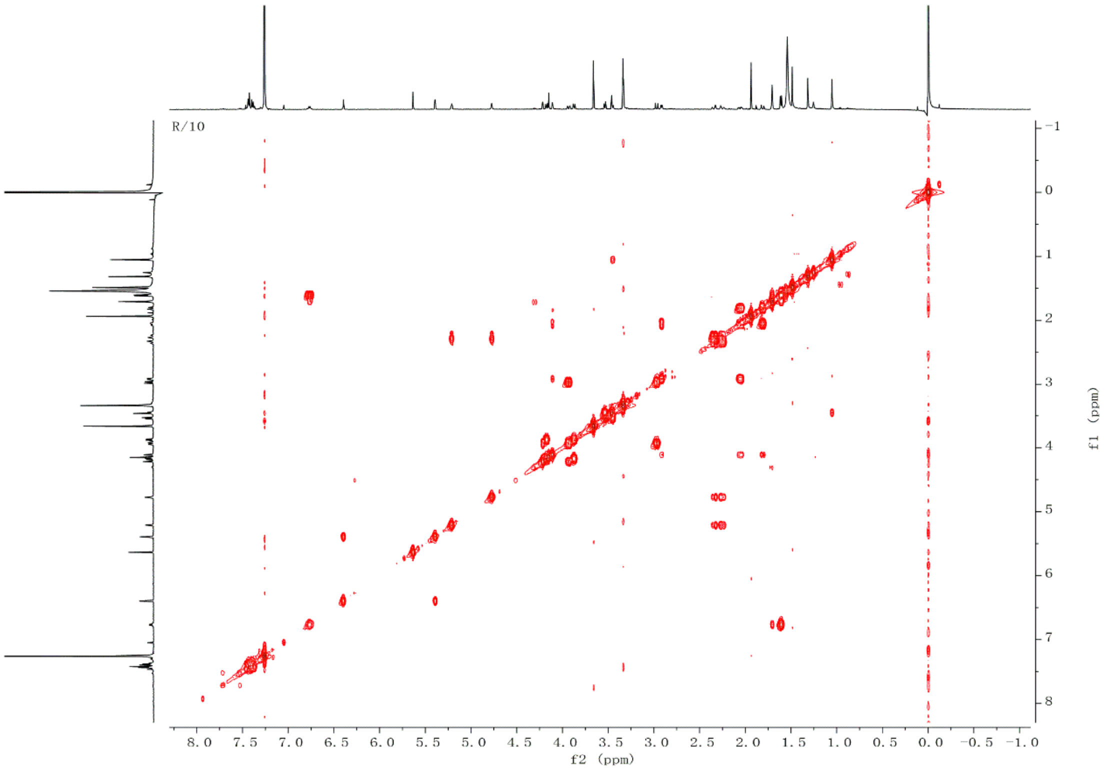

Figure S12. HRESIMS spectrum of **1b**

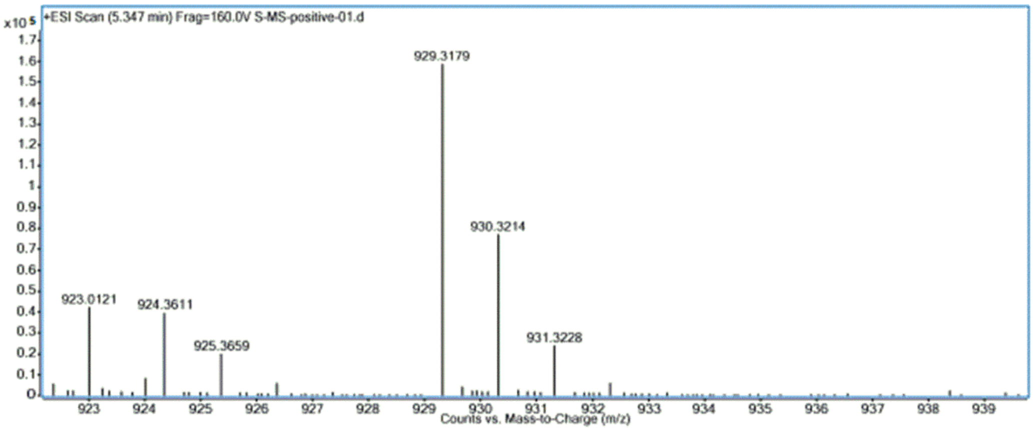

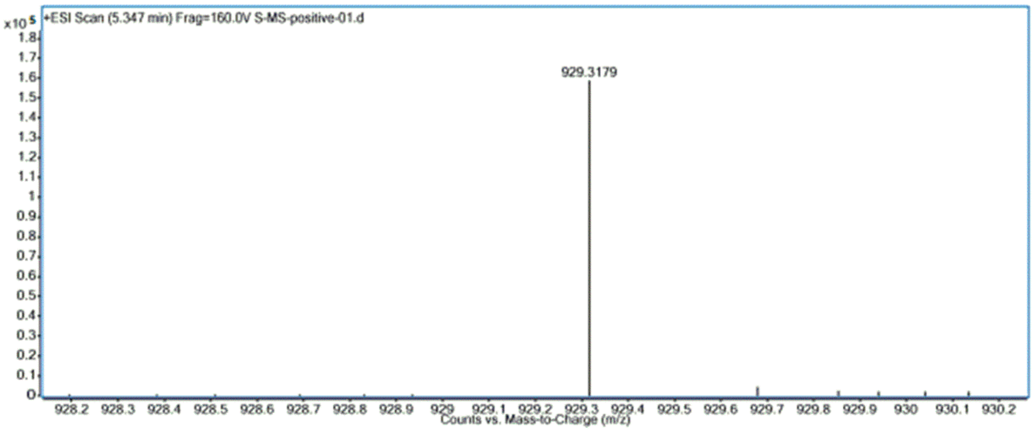

Elemental Composition Calculator

**Target m/z:**	929.3179	**Result type:**	Positive ions	**Species:**	$[M+Na]^+$
**Elements:**		C (0-80); H (0-120); O (0-30); Na (0-5)			
**Ion Formula**		**Calculated m/z**		**PPM Error**	
C45H53F3NaO16		929.3178		-0.13	

Figure S13. ^{1}H NMR (500 MHz, $CDCl_3$) spectrum of **1b**

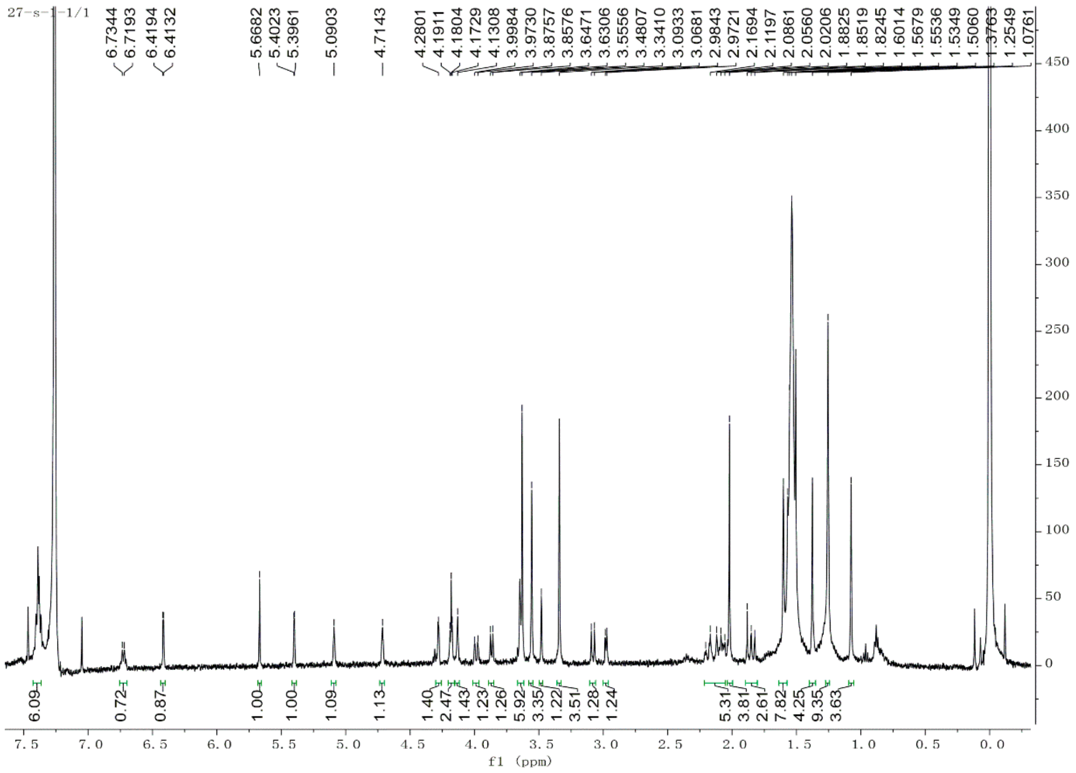

Figure S14. ROESY spectrum of **1b**

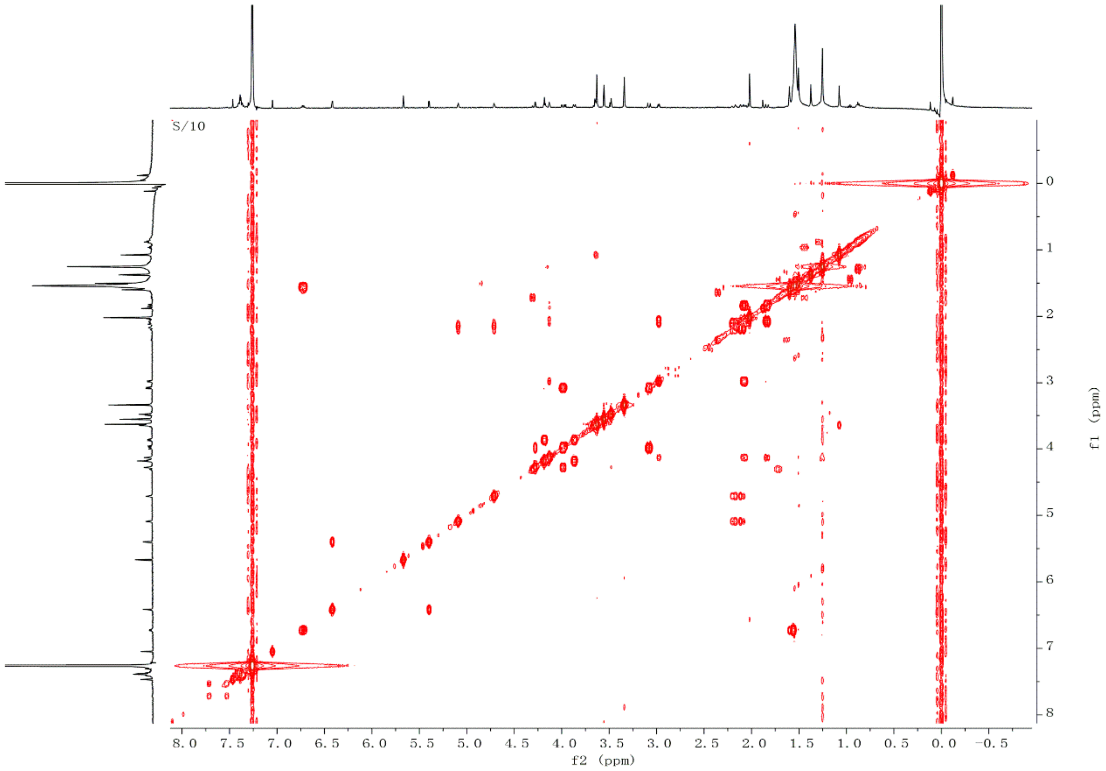

Figure S15. HRESIMS spectrum of Toosendane B (**2**)

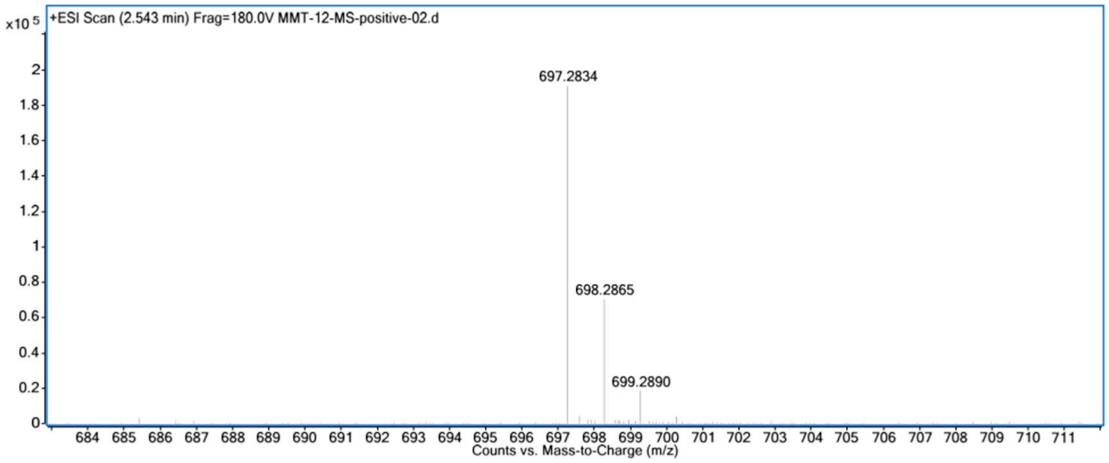

Elemental Composition Calculator

**Target m/z:**	697.2834	**Result type:**	Positive ions	**Species:**	[M+Na]+
**Elements:**		C (0-80); H (0-120); O (0-30); Na (0-5)			
**Ion Formula**		**Calculated m/z**		**PPM Error**	
C35H46NaO13		697.2831		-0.44	

Figure S16. ^{1}H NMR (500 MHz, $CDCl_3$) spectrum of Toosendane B (**2**)

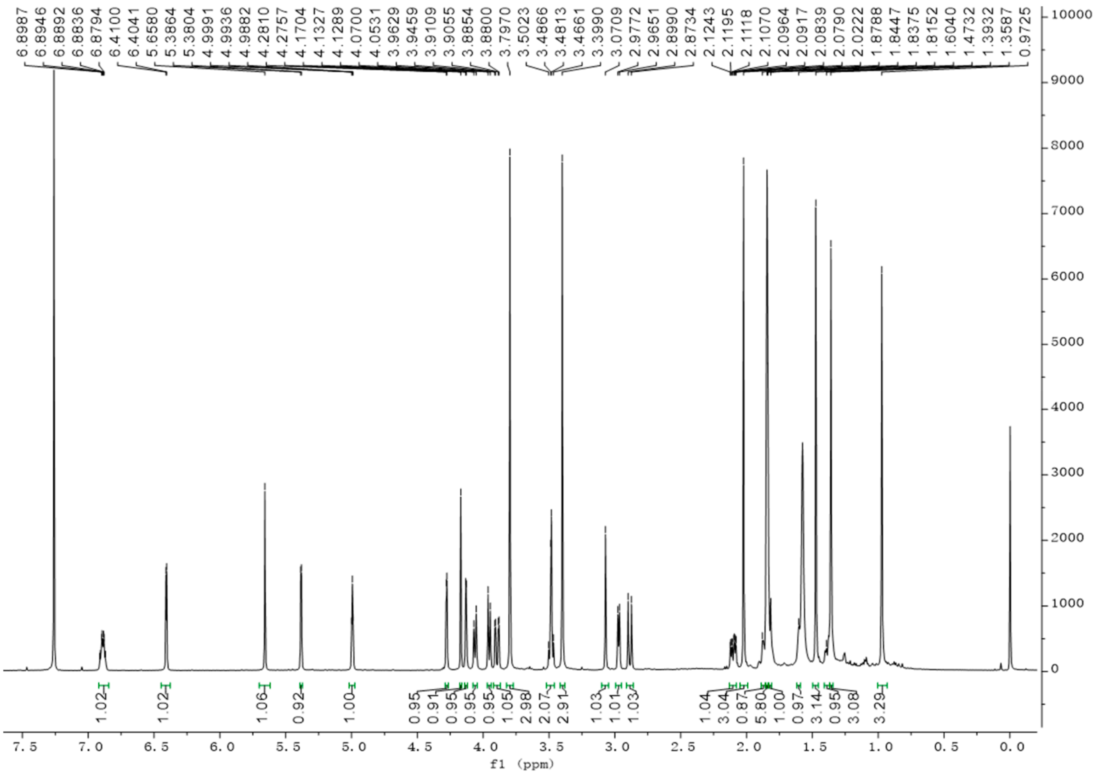

Figure S17. ^{13}C NMR (125 MHz, $CDCl_3$) spectrum of Toosendane B (**2**)

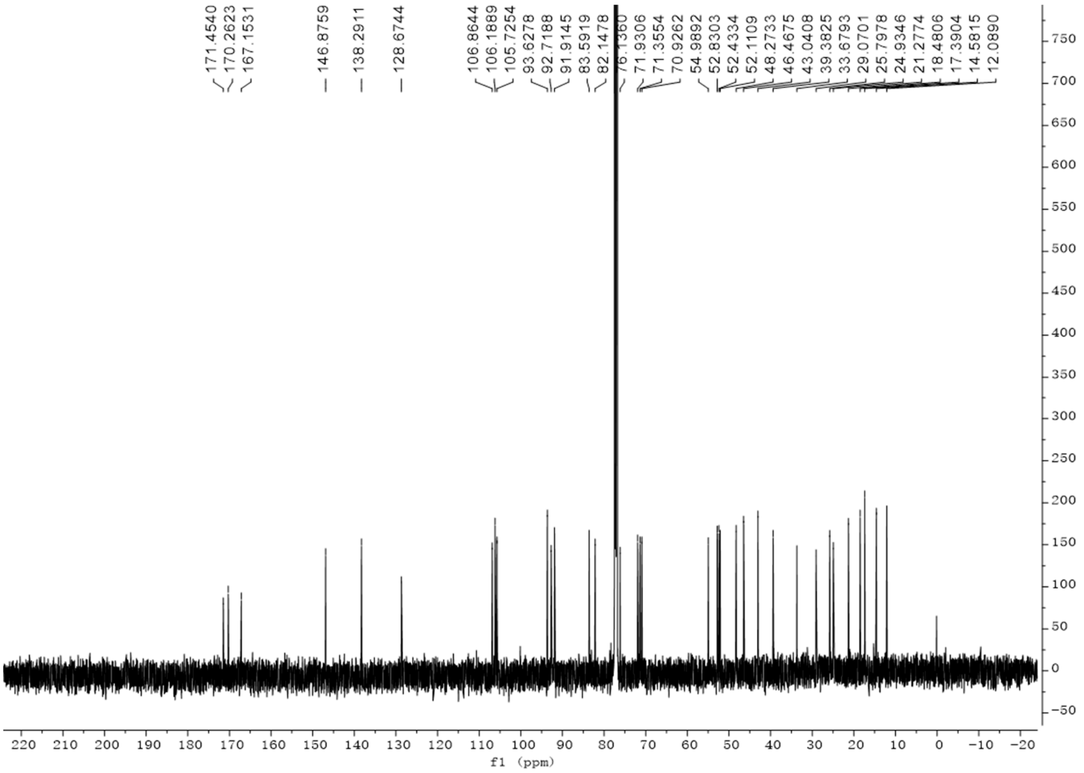

Figure S18. HSQC spectrum of Toosendane B (**2**)

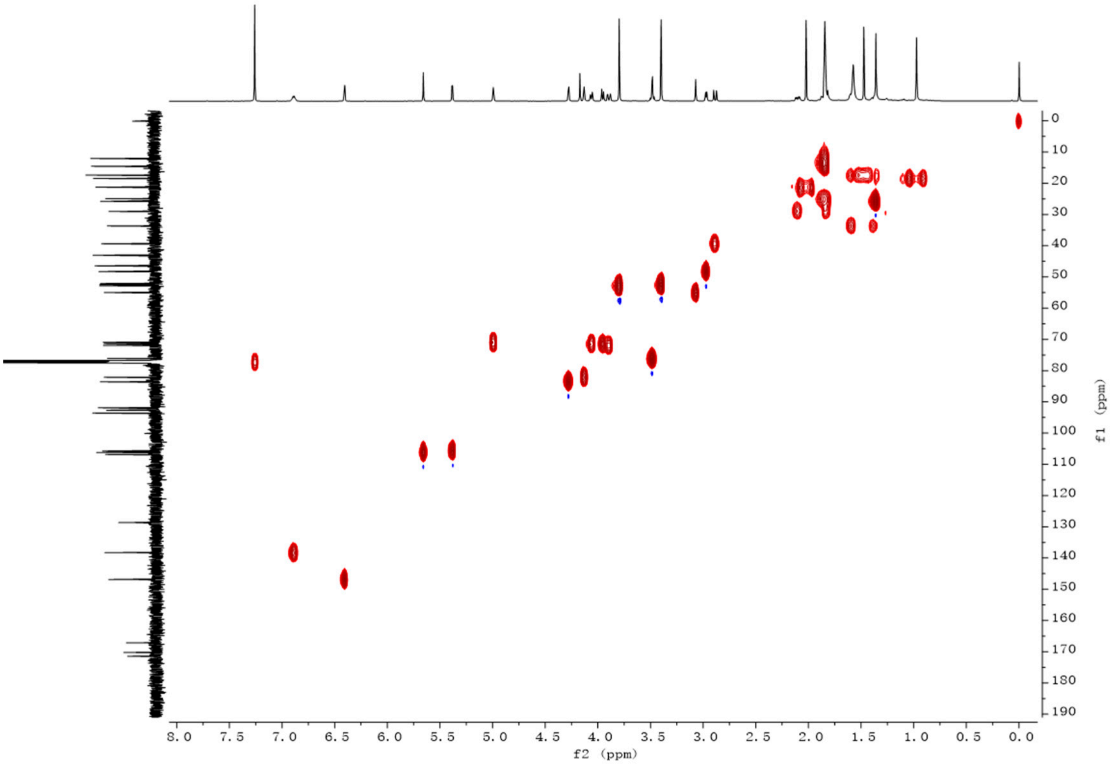

Figure S19. HMBC spectrum of Toosendane B (**2**)

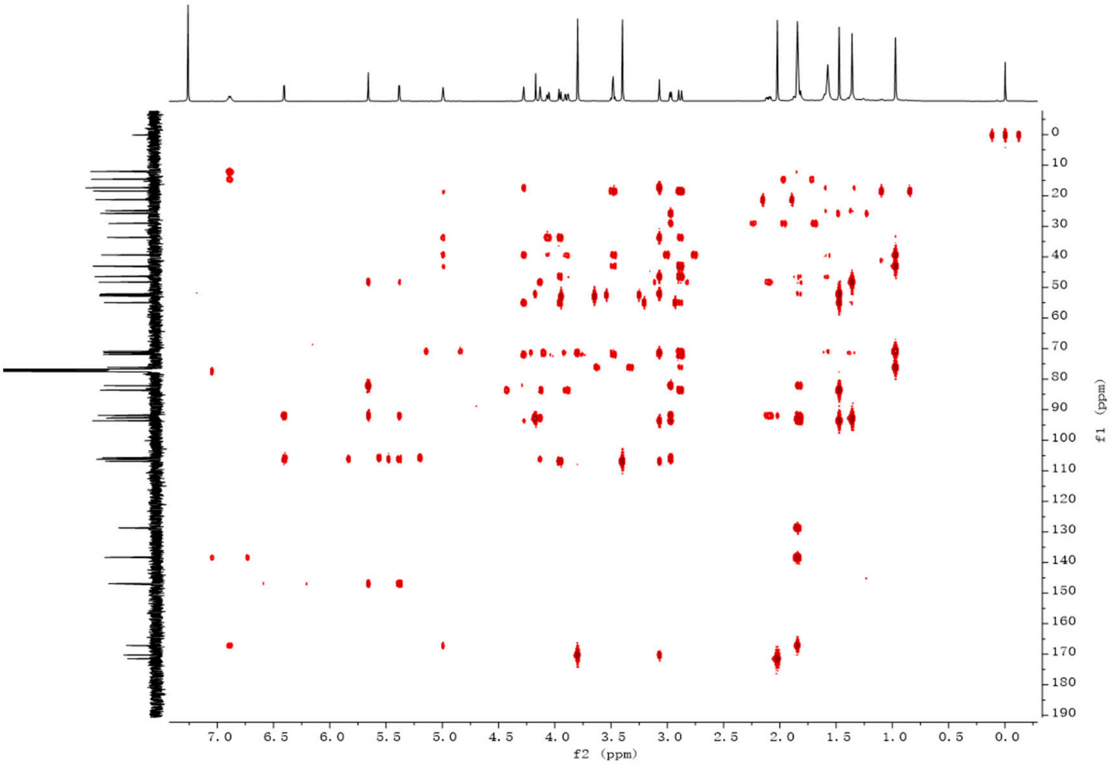

Figure S20. ROESY spectrum of Toosendane B (**2**)

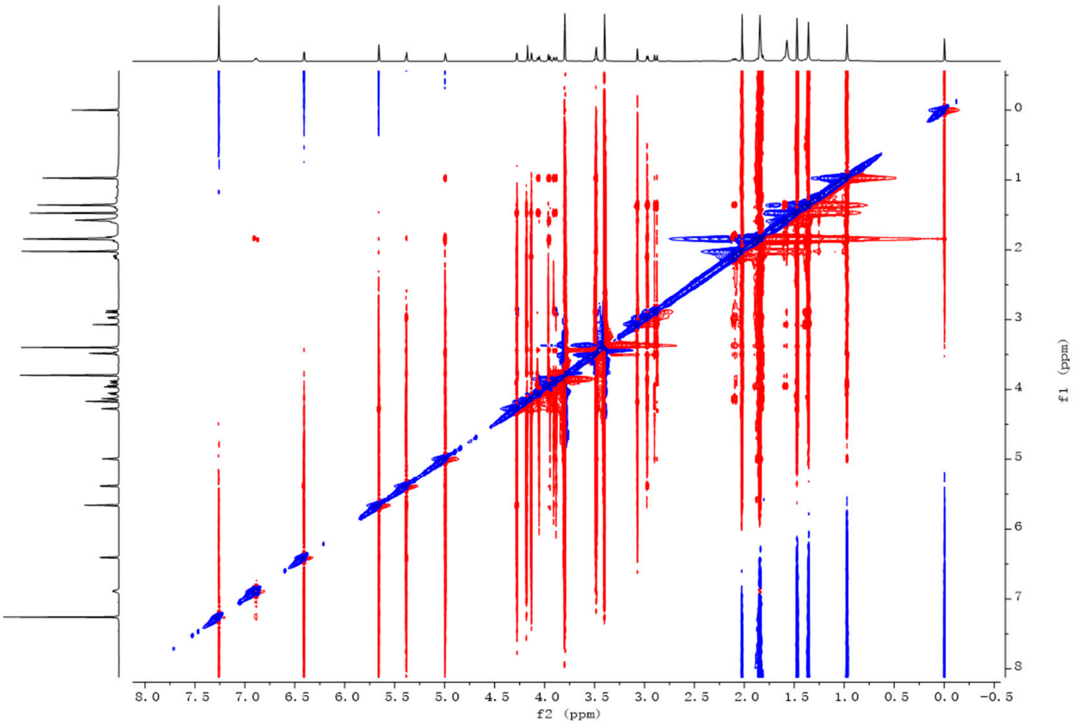

Figure S21. IR spectrum (KBr disc) of Toosendane B (**2**)

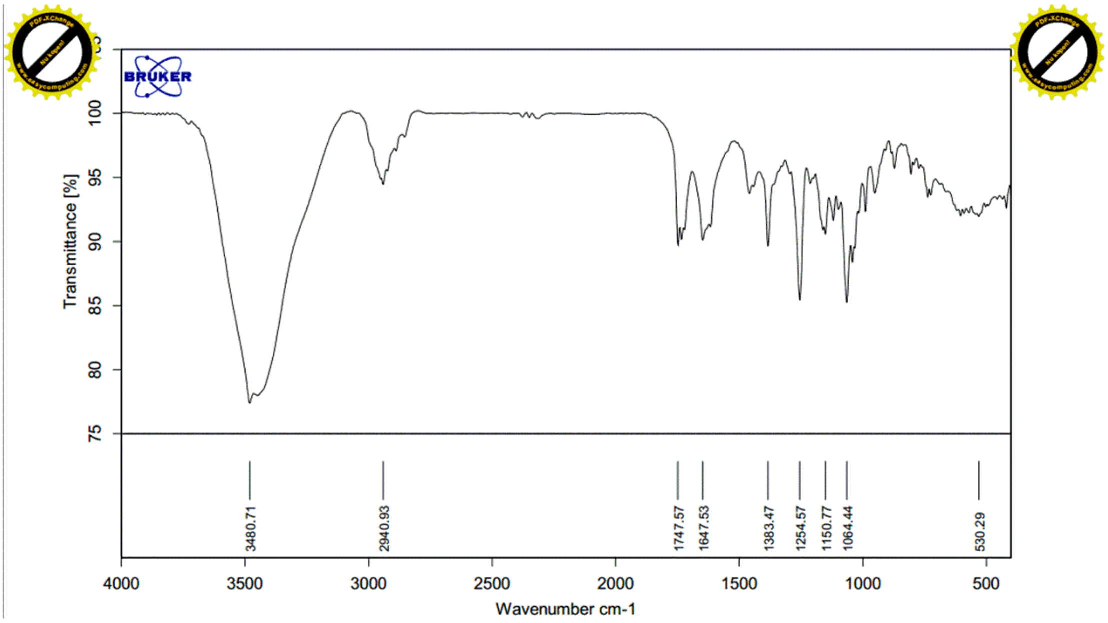

Figure S22. ECD spectra of Toosendane B (**2**) (in MeOH)

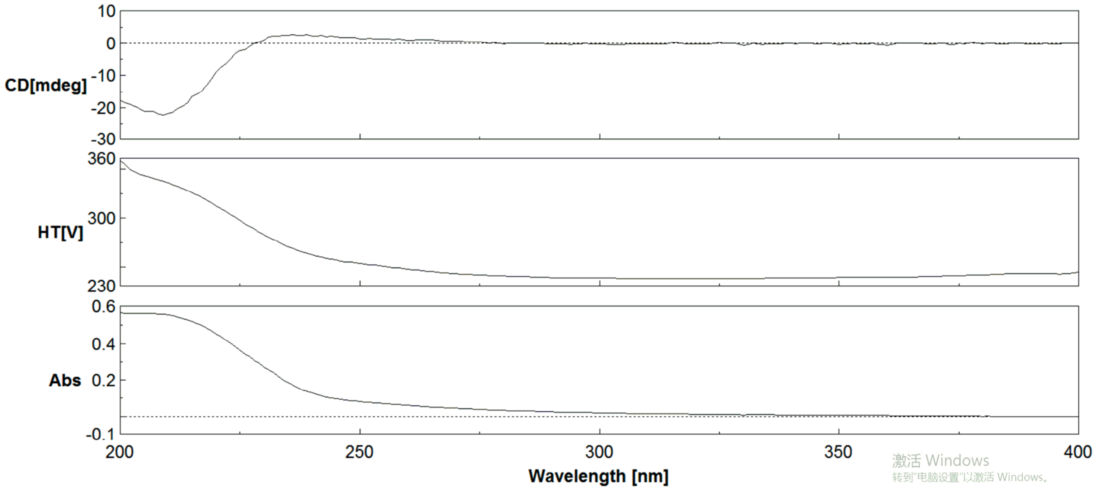

Figure S23. HRESIMS spectrum of Toosendane C (3)

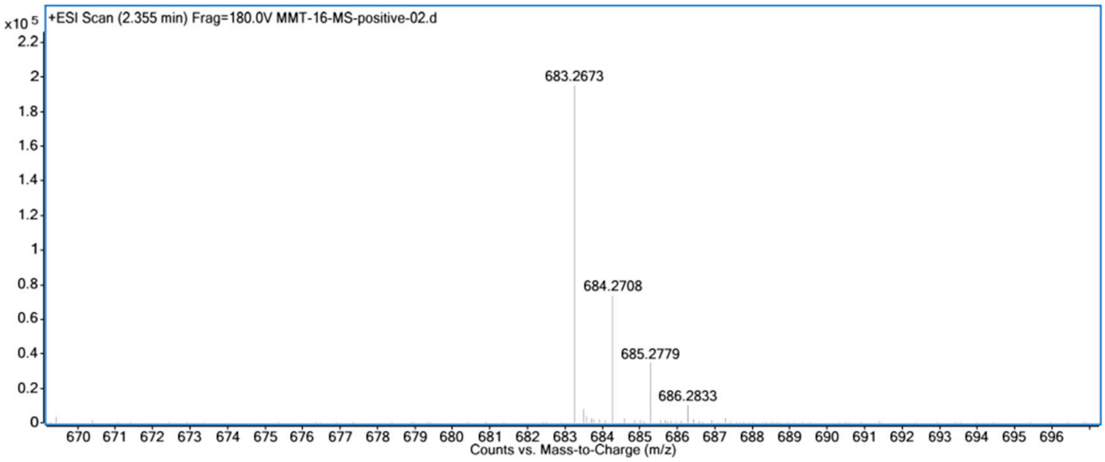

Elemental Composition Calculator

**Target m/z:**	683.2673	**Result type:**	Positive ions	**Species:**	$[M+Na]^+$
**Elements:**		C (0-80); H (0-120); O (0-30); Na (0-5)			
**Ion Formula**		**Calculated m/z**		**PPM Error**	
C34H44NaO13		683.2674		0.12	

Figure S24. ^1H NMR (500 MHz, $CDCl_3$) spectrum of Toosendane C (**3**)

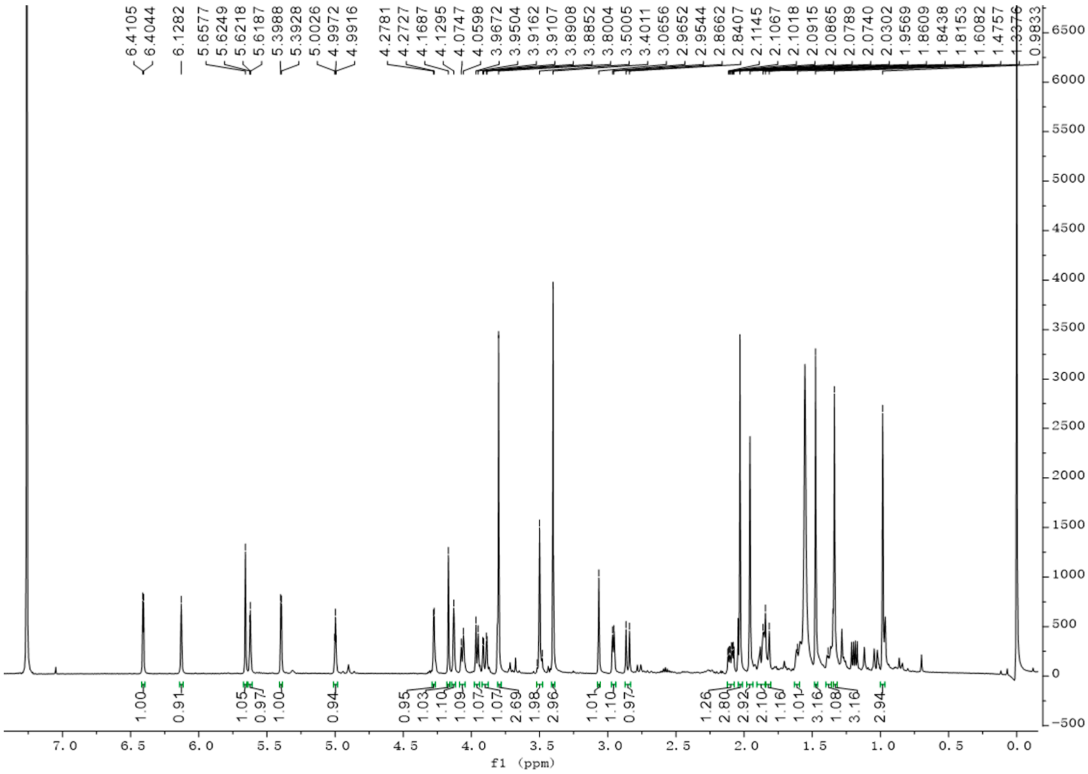

Figure S25. ^{13}C NMR (125 MHz, $CDCl_3$) spectrum of Toosendane C (**3**)

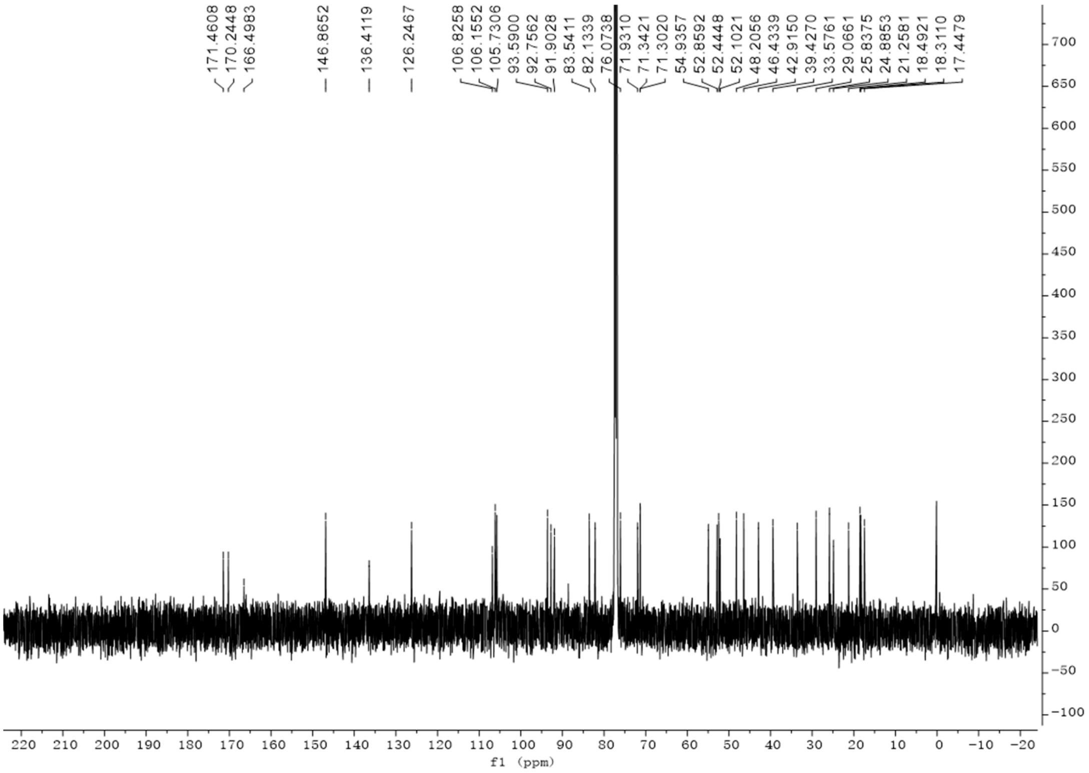

Figure S26. HSQC spectrum of Toosendane C (**3**)

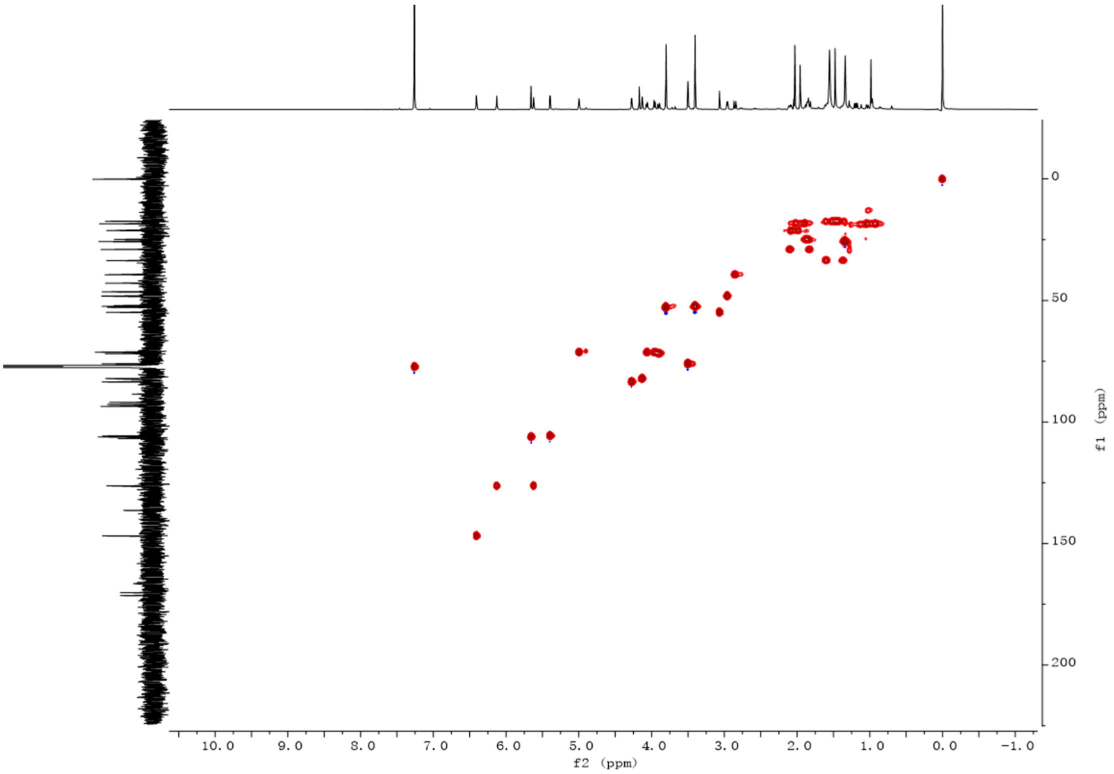

Figure S27. HMBC spectrum of Toosendane C (**3**)

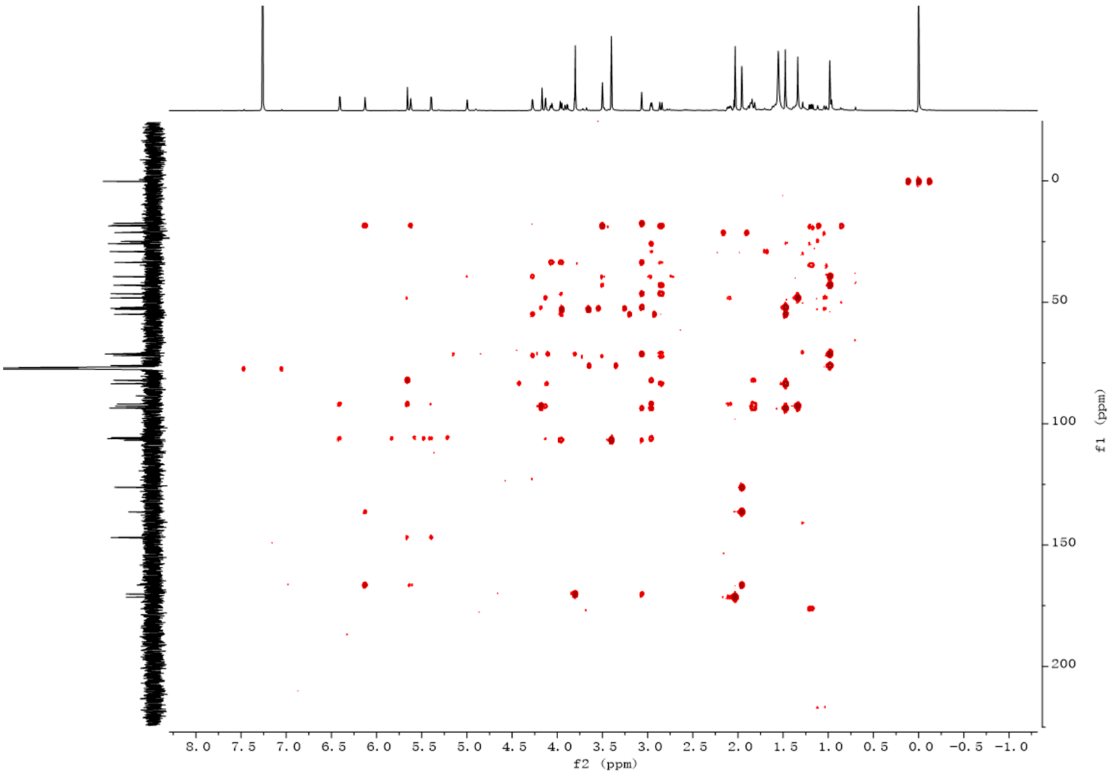

Figure S28. ROESY spectrum of Toosendane C (**3**)

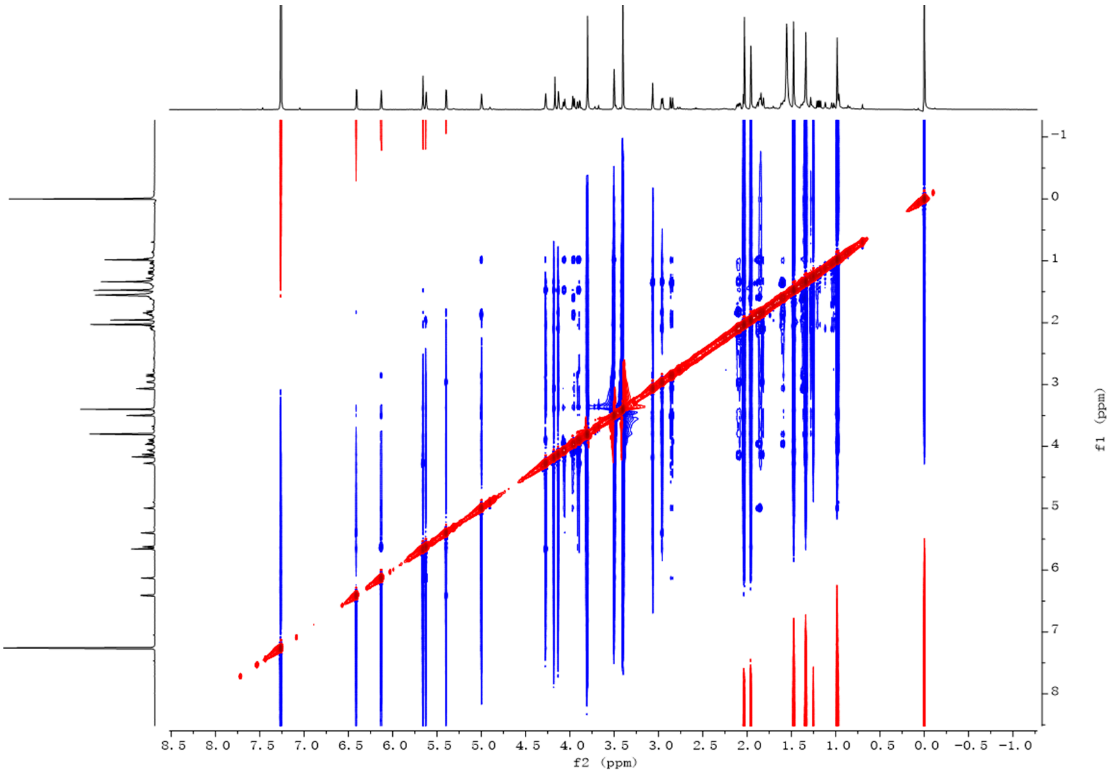

Figure S29. IR spectrum (KBr disc) of Toosendane C (**3**)

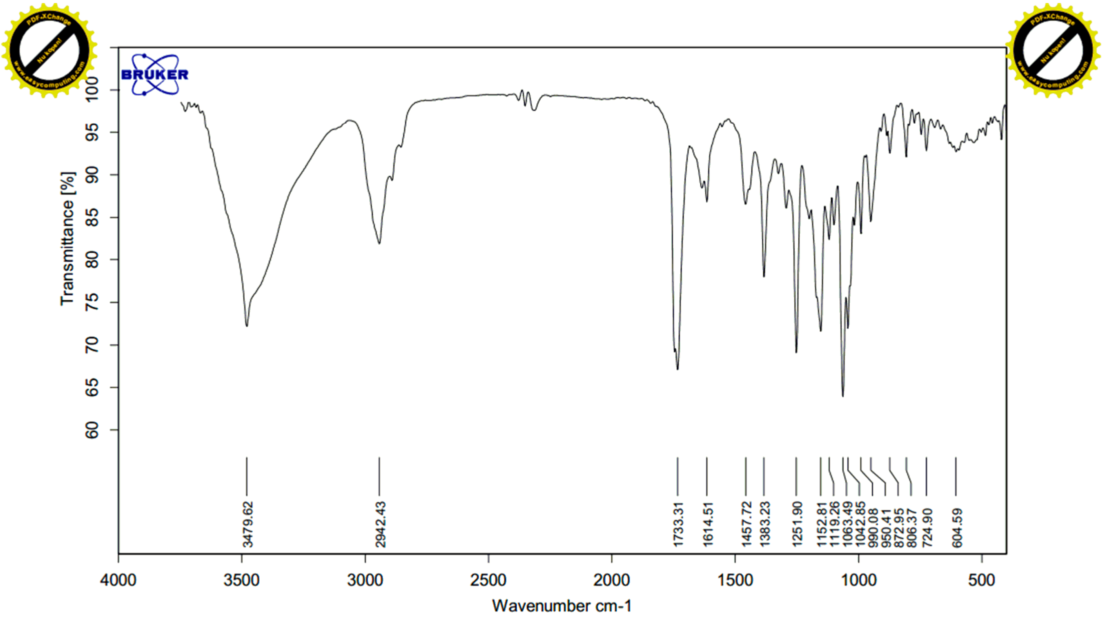

Figure S30. ECD spectra of Toosendane C (**3**) (in MeOH)

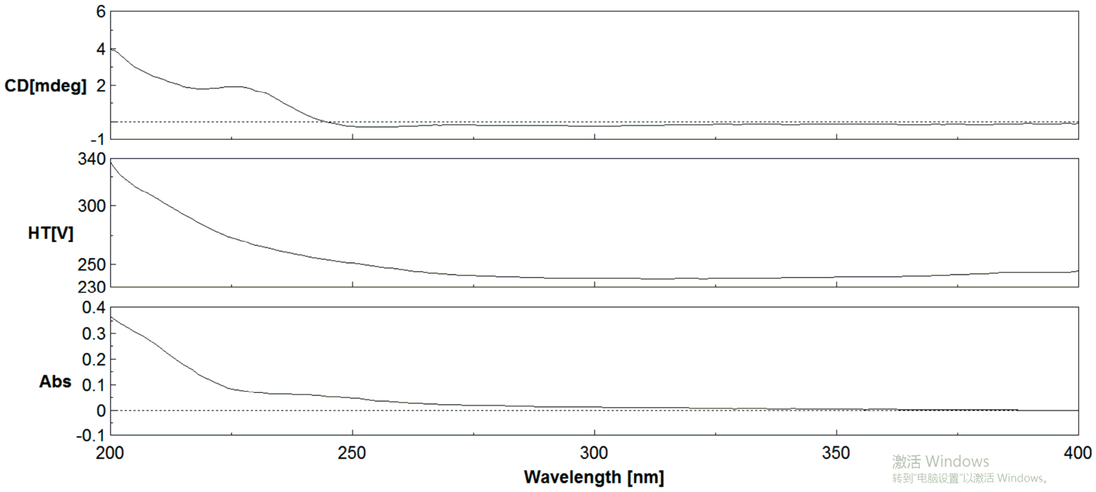

Figure S31. The NO inhibition rate and cell viabilities of toosendane B (**2**) and toosendane C (**3**) in different concentration

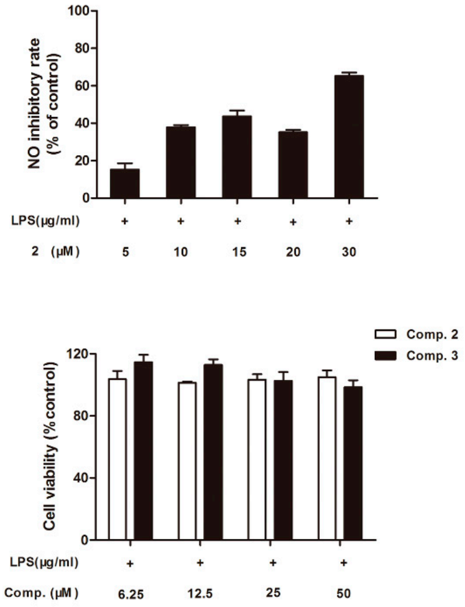

Supplement: Supplementary file 1 [file molecules-23-02590-s001.pdf]
